# Nanomaterials to combat SARS-CoV-2: Strategies to prevent, diagnose and treat COVID-19

**DOI:** 10.3389/fbioe.2022.1052436

**Published:** 2022-11-25

**Authors:** Agustín Valenzuela-Fernández, Romina Cabrera-Rodriguez, Laura Ciuffreda, Silvia Perez-Yanes, Judith Estevez-Herrera, Rafaela González-Montelongo, Julia Alcoba-Florez, Rodrigo Trujillo-González, Diego García-Martínez de Artola, Helena Gil-Campesino, Oscar Díez-Gil, José M. Lorenzo-Salazar, Carlos Flores, Jonay Garcia-Luis

**Affiliations:** ^1^ Laboratorio de Inmunología Celular y Viral, Unidad de Farmacología, Sección de Medicina, Facultad de Ciencias de la Salud, Universidad de La Laguna, San Cristóbal de La Laguna, Spain; ^2^ Research Unit, Hospital Universitario N. S. de Candelaria, Santa Cruz de Tenerife, Spain; ^3^ Genomics Division, Instituto Tecnológico y de Energías Renovables, Santa Cruz de Tenerife, Spain; ^4^ Servicio de Microbiología, Hospital Universitario N. S. de Candelaria, Santa Cruz de Tenerife, Spain; ^5^ Departamento de Análisis Matemático, Facultad de Ciencias, Universidad de La Laguna, Santa Cruz de Tenerife, Spain; ^6^ CIBER de Enfermedades Respiratorias, Instituto de Salud Carlos III, Madrid, Spain; ^7^ Faculty of Health Sciences, University of Fernando Pessoa Canarias, Las Palmas de Gran Canaria, Spain

**Keywords:** nanoreagents, personal protective equipment, vaccines, lipid nanoparticles, modified mRNA, recombinant proteins, surveillance and diagnosis, genome sequencing

## Abstract

The severe acute respiratory syndrome coronavirus 2 (SARS-CoV-2) infection and the associated coronavirus disease 2019 (COVID-19), which severely affect the respiratory system and several organs and tissues, and may lead to death, have shown how science can respond when challenged by a global emergency, offering as a response a myriad of rapid technological developments. Development of vaccines at lightning speed is one of them. SARS-CoV-2 outbreaks have stressed healthcare systems, questioning patients care by using standard non-adapted therapies and diagnostic tools. In this scenario, nanotechnology has offered new tools, techniques and opportunities for prevention, for rapid, accurate and sensitive diagnosis and treatment of COVID-19. In this review, we focus on the nanotechnological applications and nano-based materials (i.e., personal protective equipment) to combat SARS-CoV-2 transmission, infection, organ damage and for the development of new tools for virosurveillance, diagnose and immune protection by mRNA and other nano-based vaccines. All the nano-based developed tools have allowed a historical, unprecedented, real time epidemiological surveillance and diagnosis of SARS-CoV-2 infection, at community and international levels. The nano-based technology has help to predict and detect how this Sarbecovirus is mutating and the severity of the associated COVID-19 disease, thereby assisting the administration and public health services to make decisions and measures for preparedness against the emerging variants of SARS-CoV-2 and severe or lethal COVID-19.

## 1 Introduction

In the last days of the year 2019, Chinese Health Authorities reported a cluster of twenty seven patients affected with “pneumonia of unknown etiology” (seven presenting sever symptoms), which appeared to be epidemiologically associated to a seafood and wet animal wholesale market in the city of Wuhan (Hubei Province, China) (Anderson et al., 2020; Bangaru et al., 2020; Cao et al., 2020). The microorganism responsible of these “pneumonia of unknown etiology” was rapidly identified in bronchoalveolar lavage samples collected from three patients of the Wuhan Jinyintan Hospital (30 December 2019) (Cao et al., 2020). In this regard, the first named 2019-nCoV virus (2019 novel coronavirus), later on named SARS-CoV-2 (severe acute respiratory syndrome coronavirus virus 2) (ICTV, 2020), was identified by local hospitals using a surveillance mechanism for “pneumonia of unknown etiology”. This mechanism was already established in the 2003 SARS outbreak crisis with the aim of allowing timely identification of novel pathogens and reaction in future outbreaks (Cao et al., 2020; International-Committee-on-Taxonomy-of-Viruses, 2020). Importantly, this surveillance system allowed the identification of some cases that were epidemiologically unrelated to Wuhan market outbreak (Ackermann et al., 2020; Ahmed et al., 2020; Bangaru et al., 2020; Cao et al., 2020; International-Committee-on-Taxonomy-of-Viruses, 2020).

The associated illness was first named “novel coronavirus-infected pneumonia” (NCIP) and the World Health Organization (WHO) recommended that the interim name of the agent causing the current outbreak should be 2019-nCoV and was considered the causative agent of the “pneumonia of unknown etiology” (Cao et al., 2020; Shimabukuro, 2021). This name complied with the WHO Best Practices for Naming of New Human Infectious Diseases, which were developed through a consultive process among partner agencies. On 30 January 2020, WHO declared that coronavirus disease 2019 (COVID-19) was a “public-health emergency of international concern (PHEIC)” (Amaechi et al., 2020). The name of the disease was provided by the WHO International Classification of Diseases (ICD; on 11 February 2020) as COVID-19, and the official name of the virus was given by the International Committee on Taxonomy of Viruses (ICTV; on 11 February 2020), as SARS-CoV-2 (ICTV, 2020). On 11 March 2000, the WHO declared the SARS-CoV-2/COVID-19 outbreak a global pandemic (Cucinotta and Vanelli, 2020; Shimabukuro, 2021).

The first complete sequences of the new CoV (SARS-CoV-2) genome obtained from Wuhan’s patients were submitted to GISAID (Global Initiative on Sharing All Influenza Data; https://www.gisaid.org/; (Liu et al., 2020d)) (Cao et al., 2020; Lu et al., 2020) and GenBank (accession number MN908947) where the virus strain was designated as Wuhan-Human 1 CoV (Wuhan-Hu-1 or WHCV) with a whole genome sequence of 29,903 nucleotides (nt) (Ackermann et al., 2020). At the same time, it was reported the epidemiological data of nine inpatients, from at least three hospitals in Wuhan, who were diagnosed with viral pneumonia of unidentified cause (Barton et al., 2020). Furthermore, some early cases were not epidemiologically associated with the market of Wuhan (Ahmed et al., 2020; Bangaru et al., 2020; International-Committee-on-Taxonomy-of-Viruses, 2020). The sequencing of bronchoalveolar lavage fluid samples of the above nine patients again identified the new CoV (Barton et al., 2020). More evidence for the presence of this CoV was obtained by virus isolation form the clinical specimens, viral culture, and cytotoxicity assays together with virus morphology analysis by electron microscopy (Barton et al., 2020; Cao et al., 2020), all of which are methods and devices that clearly use key nanotechnologies for SARS-CoV-2/COVID-19 detection and diagnosis (Caldas et al., 2020; Prasad et al., 2020; Akilesh et al., 2021; Jefferson et al., 2021; Sung et al., 2022).

**FIGURE 1:**
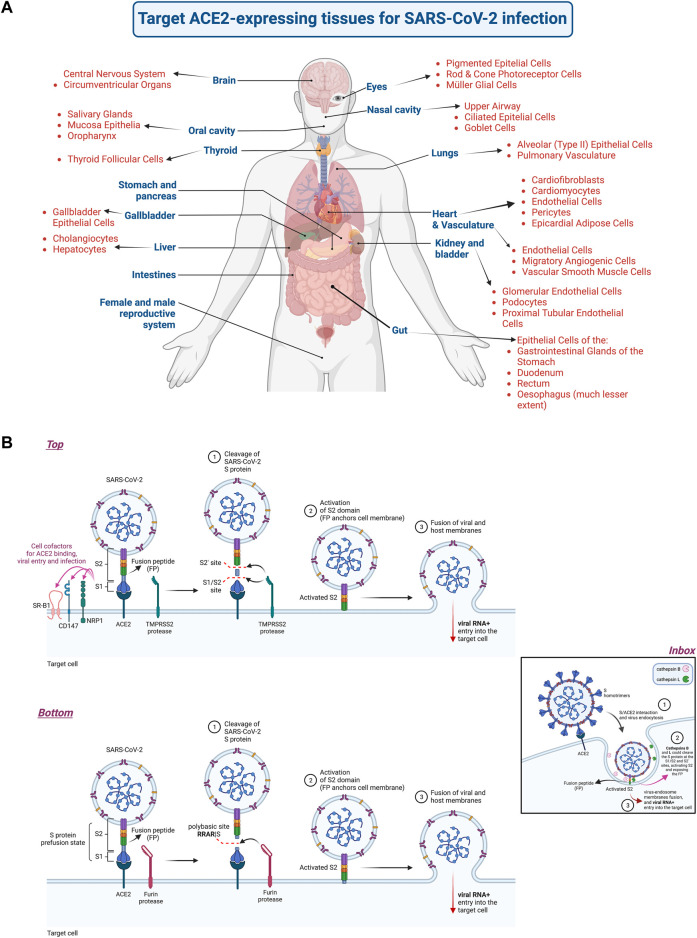
Cells, tissues and organs that express the ACE2 receptor and are infected by SARS-CoV-2, and the mechanisms of early SARS-CoV-2 infection. **(A)** Scheme representing cells, tissues and organs expressing the ACE2 receptor that allows the SARS-CoV-2 virus to infect them, thereby being associated with tissue damage and organ failure. **(B)** Scheme representing the main viral and cellular molecules involved in SARS-CoV-2 attachment and fusion with target cell that mediate viral entry and infection. The S protein (with its S1 and S2 subunits in the prefusion state) interacts with ACE2 leading to conformational changes in the protein that facilitate the proteolytic cleavage of the S protein that further exposes the fusion peptide (FP). This S cleavage is catalyzed by several host proteases, such as TMPRSS2 that recognizes the canonical S1/S2 cleavage site, as well as other sites at the S protein (e.g., S2’) (*Top scheme*), and furin that excises at the polybasic region (RRAR|S) located between S1 and S2 subunits, and near the S2-fusion peptide (*Bottom scheme*). The S protein could also be cleaved by the endosome enzymes cathepsin B/L, after ACE2-mediated endocytosis of the virus, promoting virus-endosome membranes fusion (*Inbox scheme*). All these proteolytic cleavages expose the fusion peptide (FP) of the S2 subunit of the virus, facilitating virus-cell membranes fusion and the entry of viral RNA + genome into the cell, starting the SARS-CoV-2 infection process. Several cofactors have been reported to be involved in SARS-CoV-2 entry and infection, such as NRP1, reported to mediate SARS-CoV-2 entry into the brain via the olfactory bulb, the SR-B1 receptor, proposed to promote SARS-CoV-2 entry (in an ACE2-dependent manner) and the transmembrane glycoprotein CD147 (BSG or EMMPRIN) that could mediate viral entry and infection by endocytosis cooperating with ACE2 (*Top scheme*). Designs and templates are created with BioRender.

The disease symptoms vary from person to person, from a paucisymptomatic or mild respiratory illness to an acute distress respiratory syndrome (ARDS). However, the prevalent reported symptoms associated with SARS-CoV-2/COVID-19 are fever, cough, fatigue, dyspnea (shortness of breath) and anosmia (partial or complete loss of the sense of smell), sore throat, headaches, ocular manifestations, chest pain and diarrhea. It also includes abnormal coagulation and lymphopenia that together with cytokine storm, hyper-inflammation, sepsis and septic shock accompany severe pneumonia, ARDS and multiorgan dysfunction, including neuropathogenesis, liver, kidney and heart failure (Bizzarro et al., 2011; Ackermann et al., 2020; Ahmed et al., 2020; Amaechi et al., 2020; Arslan et al., 2020; Baig et al., 2020; Bangaru et al., 2020; Barton et al., 2020; Brann et al., 2020; Cao et al., 2020; Chan et al., 2020; Chen et al., 2020; Cheung et al., 2020; China-National-Health-Commission-(Beijing), 2020; Corbett et al., 2020; Devaux et al., 2020; Diao et al., 2020; Gavriatopoulou et al., 2020; Gibson et al., 2020; Goyal et al., 2020; Guan et al., 2020; Hariri and Hardin, 2020; Helms et al., 2020; International-Committee-on-Taxonomy-of-Viruses, 2020; Kuster et al., 2020; Lechien et al., 2020; Ledford, 2020; Levi et al., 2020; Lipsitch et al., 2020; Mazzoni et al., 2020; Menter et al., 2020; Ouyang et al., 2020; Puelles et al., 2020; Ruan et al., 2020; Seah and Agrawal, 2020; Toscano et al., 2020; Tsivgoulis et al., 2020; Verdoni et al., 2020; Baden et al., 2021; Shimabukuro, 2021). Therefore, COVID-19 infections affect various organs in the body, including the central nervous system (Baig et al., 2020; Wu et al., 2020), cardiovascular system (Zheng et al., 2020), kidneys (Cheng et al., 2020b), gastrointestinal tract (Wong et al., 2020b), and liver (Zhang et al., 2020a; Bangash et al., 2020) (Figure 1A). Furthermore, a common cause of death is an uncontrolled hyperinflammatory response (i.e., with uncontrolled cytokine levels), which can lead to stroke due to blood clots, organ failure, and heart attacks (Hasanzadeh et al., 2021). Since the SARS-CoV-2 must bind to angiotensin-converting enzyme 2 (ACE2) to enter and infect host cells in humans (Zhou et al., 2020a; Shang et al., 2020b; Walls et al., 2020b; Wang et al., 2020e; Lan et al., 2020), the expression (partly determined by the stage of development) and body localization of ACE2 are important to understand COVID-19 symptoms and disease (Hamming et al., 2004; Yang et al., 2020b; Ni et al., 2020) (Figure 1A). Noteworthy, ACE2 is a zinc metallopeptidase involved in regulation of blood pressure and cardiac function (Tikellis and Thomas, 2012). It has recently been reported that SARS-CoV-2-targeted ACE2-expressing organs lead to severe pathobiological manifestations followed by multiple organ failure (Salamanna et al., 2020) (Figure 1A, *scheme representing the different organs and tissues that express ACE2, and could be infected by SARS-CoV-2*). The ACE2 receptor for SARS-CoV-2 infection was previously reported to be the receptor for the Sarbecovirus SARS-CoV-1 (Li et al., 2003) and for the human coronavirus (HCoV)-NL63 (Hofmann et al., 2005), an alphacoronavirus known to cause mild upper respiratory tract infection. The entry steps of the SARS-CoV-2 viral particles are mediated by the external S glycoprotein, which is arranged as a homotrimers (peplomers) (Cavanagh, 1995) that give the characteristic Sun’s corona-like aspect to the viral particles (Liu et al., 2020b; Liu et al., 2020c) (see images at NIH (NIAID) website “https://www.flickr.com/photos/niaid/albums/72157712914621487”), therefore classified as the *coronaviridae* family (de Groot et al., 1987). SARS-CoV-2 fusion with cell-plasma membrane and cell entry are therefore initiated by the interaction of the receptor binding domain (RBD) of the S protein to human ACE2 at the cell-surface (Li et al., 2003; Li et al., 2005; Yan et al., 2020a; Wang et al., 2020e; Wan et al., 2020) (Figure 1B, *scheme representing key viral and cell molecules involved in virus attachment and fusion with target cell that mediate viral entry and infection*). Then, the S protein, anchored to ACE2 in its prefusion state (Figure 1B), undergoes proteolytic cleavage catalyzed by several host proteases, such as furin that cleaves at the polybasic region (RRAR685|S), and TMPRSS2 (transmembrane serine protease 2) that recognizes the canonical S1/S2 cleavage site, as well as other sites at the S protein (Hoffmann et al., 2020a; Bestle et al., 2020; Hoffmann et al., 2020b; Wang et al., 2020e; Fraser et al., 2022) (Figure 1B). Likewise, the S protein could be also cleaved by cathepsin B/L, within endosomes after virus ACE2-mediated endocytosis, promoting virus-endosome membrane fusion (Hoffmann et al., 2020b; Bollavaram et al., 2021; Ou et al., 2021) (Figure 1B, *Inbox scheme*). All these proteolytic cleavages expose the fusion peptide of the S2 subunit of the virus, facilitating virus-cell membranes fusion (Figure 1B) and the entry of viral RNA + genome into the cell, starting the SARS-CoV-2 infection process (reviewed in (Peng et al., 2021a; Zhang et al., 2021a; Jackson et al., 2022)). Furin could also cleave the S protein into the S1 and S2 subunits, which remain associated, during the late steps of the viral cycle in virus-producing cells (Hoffmann et al., 2020a; Shang et al., 2020a). This event leads to new viral particles that easily expose the S2-fusion peptide favoring cell-cell spread and cell infection (Hoffmann et al., 2020a; Shang et al., 2020a; Papa et al., 2021) (Figure 1B, *Bottom scheme*). The furin cleavage site at the S protein is thought to be responsible for the virulence of SARS-CoV-2 (Hoffmann et al., 2020a; Johnson et al., 2020; Johnson et al., 2021). Furthermore, several cofactors have been involved in SARS-CoV-2 entry and infection (Figure 1B, *Top scheme*). Thus, Neuropilin-1 (NRP1) appears to trigger SARS-CoV-2 viral entry and infection (Cantuti-Castelvetri et al., 2020; Daly et al., 2020). NRP1 has been proposed to mediate SARS-CoV-2 entry into the brain via the olfactory bulb (Cantuti-Castelvetri et al., 2020), since NRP1 is expressed in respiratory and olfactory epithelia (Cantuti-Castelvetri et al., 2020; Daly et al., 2020). Moreover, high-density lipoprotein (HDL) scavenger receptor B type 1 (SR-B1) has been reported to promote SARS-CoV-2 entry, in an ACE2-dependent manner (Wei et al., 2020), whereas the transmembrane glycoprotein CD147 (also named BSG (basigin (Ok blood group) (Kaname et al., 1993) or EMMPRIN (extracellular matrix metalloproteinase inducer) (Biswas et al., 1995)), could mediate viral entry and infection by endocytosis, even in cells lacking ACE2 expression (Wang et al., 2020c). Moreover, it seems that CD147 and ACE2 cooperate during SARS-CoV-2 infection, since ACE2 levels are modulated by CD147 density and they are coregulated by viral infection (Fenizia et al., 2021).

Therefore, nano-based strategies to battle SARS-CoV-2/COVID-19 must confront all symptoms of the disease, particularly to avoid severe outcomes, and to neutralize the infection of cells from different tissues and organs that could be targeted by the virus, through vaccine-elicited specific and optimal immune responses, mainly driven against the S viral immunogen.

In this scenario, viral isolation, culture and sequencing were key methods, based on nanotechnology and nano-reagents or materials that help to achieve fasts diagnosis and to endorse and develop urgent public health measures and responses, as the WHO preparedness for emerging epidemic threats recommends (McCloskey et al., 2014; Elbe and Buckland-Merrett, 2017; Jain et al., 2018; Kluge et al., 2018; Global-Preparedness-Monitoring-Board-(GPMB), 2019; Baden et al., 2021). These nano-based tools and strategies allowed the detection of a new RNA+ genome from patients’ samples, and the identification of the new SARS-CoV-2 virus of the lineage B of the genus betacoronavirus (Cao et al., 2020). In general, nanomaterials and nanotechnological approaches to combat viral infections consist in diagnostic sensors, vaccine formulations, and protective coatings, and vehicles for delivery of anti-viral vaccines and adjuvants, as well as for new treatments to palliate COVID-19 associated symptoms and to limit disease severity (reviewed in (Campos et al., 2020; Cavalcanti and Cajubá De Britto Lira Nogueira, 2020; Chung et al., 2020; Gupta et al., 2020; Pisani et al., 2020; Talebian and Conde, 2020; Weiss et al., 2020; Asdaq et al., 2021; Constantin et al., 2021; Petkar et al., 2021; Rashidzadeh et al., 2021; Varahachalam et al., 2021; Goharshadi et al., 2022; Souri et al., 2022)). Nanoreagents and derived materials have intrinsic antipathogenic properties that are able to inactivate and degrade the SARS-CoV-2 virus, as well as other viruses, bacteria and microorganisms via generation of reactive oxygen species in contact with the virus or triggered by photocatalysis. We have considered materials used in face masks and protective personal equipment (PPE) for prevention. These nanomaterials could offer new tools and disinfection methods in healthcare facilities. Vehicles used for drug delivery are similar to those used for vaccine-associated immunogen delivery, which could function as adjuvants for immune system activation (reviewed in (Petkar et al., 2021)). We have considered nano-based vaccines as the real treatment for prevention and treatment of SARS-CoV-2 infection and COVID-19 disease. We have summarized the main diagnoses tests used (serological and RT-qPCR, useful in point-of-care facilities) (Valera et al., 2021) and emphasizing genome-mediated approximations based on nanopore technology (Abraham et al., 2008; Ciuffreda et al., 2021b; Liefting et al., 2021; Lin et al., 2021; Ju et al., 2022), an outstanding electrochemical biosensor on nanoscale used for SARS-CoV-2 detection and sequencing (Yakovleva et al., 2022), and viral whole genome sequencing (WGS) (Gohl et al., 2020; Rosenthal et al., 2022). These strategies are considered high performance nanotechniques for virus genome sequencing, diagnosis and virus surveillance, where their utility to combat SARS-CoV-2/COVID-19 have been clearly demonstrated in this pandemic crisis. All these nano-based designs, tools and strategies have allowed a historical, unprecedent, real time epidemiological surveillance, diagnosis of SARS-CoV-2 infection and transmission, at community and international levels, predicting how this Sarbecovirus is mutating and the severity of the associated COVID-19 disease.

In this review we aimed to address all these nano-based strategies to battle SARS-CoV-2/COVID-19 that assist the administration and public health services to make decisions and measures for preparedness against the emerging variants of SARS-CoV-2 and severe or lethal COVID-19.

## 2 Nanomaterials in the battle against SARS-CoV-2: Physical barriers, vaccines, genomics-based virosurveillance and diagnosis

### 2.1 Physical barriers for prevention: Face masks and protective personal equipment

The entire world has witnessed that prevention strategies against the still ongoing COVID-19 pandemics have included vaccines, antiviral drugs and, since the beginning, personal protective countermeasures, such as facemasks together with physical distancing (Chu et al., 2020; Brainard et al., 2020; Li et al., 2021b; Raman et al., 2021; Rowan and Moral, 2021). In fact, nanomaterials and associated technologies are also crucial to control person-to-person virus transmission and viral community spreading (i.e., face masks, sanitizers and disinfectants, drugs and vaccines) (Barycka et al., 2020; Chu et al., 2020; Brainard et al., 2020; Carraturo et al., 2020; Ilyas et al., 2020; Asdaq et al., 2021; Li et al., 2021b; Dhama et al., 2021; Hodgson et al., 2021; Meo et al., 2021; Rohilla, 2021; Rowan and Moral, 2021; Salian et al., 2021; Tabatabaeizadeh, 2021; Thompson et al., 2021; Vitiello et al., 2021; Kianpour et al., 2022). If these measures to control transmission are neglected it will lead to the emergence of a plethora of new variants of the SARS-CoV-2 virus [including the variants of concerns (VOCs)] with may associate with increased infectious and transmission properties that compromise natural and vaccine-elicited immune responses and protection, as well as the effectiveness of the assays for rapid detection and diagnosis (Alcoba-Florez et al., 2020a; Weisblum et al., 2020; Bal et al., 2021; Bian et al., 2021; Boehm et al., 2021; Camp et al., 2021; Liu et al., 2021d; Hoffmann et al., 2021; Raman et al., 2021; Salleh et al., 2021; Singh et al., 2021; Tao et al., 2021; Alejo-Cancho et al., 2022; Mertens et al., 2022; Sonnleitner et al., 2022).

The main transmission route of SARS-CoV-2 from person to person is aerosol (Morawska and Cao, 2020; Setti et al., 2020), among respiratory droplets, oral-fecal transmission and contact (i.e., fomites), bloodborne, mother-to-child (reported to occur at low rates) and animal-to-human transmission (Ackermann et al., 2020; Ahmed et al., 2020; Amaechi et al., 2020; Arslan et al., 2020; Aydillo et al., 2020; Bangaru et al., 2020; Bonato et al., 2020; Brann et al., 2020; Cao et al., 2020; Carvalho et al., 2020; Chang et al., 2020; Cheung et al., 2020; Dona et al., 2020; Eslami and Jalili, 2020; Grassia et al., 2020; Guan et al., 2020; Guo et al., 2020c; Hoffmann et al., 2020b; Huang et al., 2020a; International-Committee-on-Taxonomy-of-Viruses, 2020; Jin et al., 2020; Khanh et al., 2020; Lechien et al., 2020; Lednicky et al., 2020; Lescure et al., 2020; MacIntyre and Ananda-Rajah, 2020; Mondelli et al., 2020; Nishiyama et al., 2020; Nzediegwu and Chang, 2020; Pastorino et al., 2020; Poggio et al., 2020; Rothe et al., 2020; Santarpia et al., 2020; Shereen et al., 2020; van Doremalen et al., 2020; Voskarides, 2020; Wang et al., 2020f; Xu et al., 2020a; Al-Sharif et al., 2021; Baden et al., 2021; Birkhead et al., 2021; Chan et al., 2021; Chaubey et al., 2021; Chaudhary et al., 2021; Conti et al., 2021; Cuicchi et al., 2021; Dumitriu et al., 2021; Kumar et al., 2021; Kutter et al., 2021; Musa et al., 2021; Peacock et al., 2021a; Sawatzki et al., 2021; Shimabukuro, 2021; Valencak et al., 2021; Yang et al., 2021a; Zaneti et al., 2021; Chan et al., 2022; Costa et al., 2022; de Oliveira-Filho et al., 2022; Jairak et al., 2022; Kang et al., 2022; Katona et al., 2022; Kyle et al., 2022; Patane et al., 2022; Pecora et al., 2022; Wake, 2022; WHO, 2022). The average size of this virus is 60–140 nm with a volume of about 10^6^ nm^3^ (Bar-On et al., 2020; Zhu et al., 2020c). The likelihood of airborne transmission is very low unless droplets (less than 5 μm in diameter) are contaminated with the virus and remain in the air for long periods. The air transports exhaled droplets that might contain the virus, when expired, the liquid content evaporates, diminishing their size. These small droplets are free to travel in the air, thereby being easily transported by air currents, carrying their viral content several meters from where they originated and occupying the entire volume of a room (Bourouiba, 2020; Setti et al., 2020). SARS-CoV-2 is highly stable in aerosol and on surfaces compared to SARS-CoV-1, maintaining the virus infectious for hours in the aerosol (Anfinrud et al., 2020; Morawska and Cao, 2020; Paules et al., 2020; van Doremalen et al., 2020; Ong et al., 2021). These facts imply that in the absence of face masks the distance of 1–2 m among people is not enough to safeguard from SARS-CoV-2 infection risk (Parshina-Kottas et al., 2020), as it has been reported to happen in hospitals (i.e., SARS-CoV-2 RNA has been detected in air samples collected inside the hospitals, thereby the airborne route has to be considered an important pathway for contamination), schools and other indoors spaces (Liu et al., 2020f; Nissen et al., 2020; Santarpia et al., 2020; Akhmetzhanov et al., 2021; Chau et al., 2021; Ding et al., 2021; Kumar et al., 2021; Kumari et al., 2021; Leeman et al., 2022; Marchese et al., 2021; Miyoshi et al., 2021; Murewanhema et al., 2021; Ong et al., 2021; Salmenjoki et al., 2021; Sami et al., 2021; San et al., 2021; Jung et al., 2022; Kirsten et al., 2022; Ladhani et al., 2022; Leng et al., 2022; Nagy et al., 2022; Polechova et al., 2022; Viner et al., 2022; White et al., 2022). Therefore, SARS-CoV-2 is transmitted by bioaerosols (<10 μm) and droplets (>10 μm) projected during breathing, speaking and coughing (Klompas et al., 2020a; Binder et al., 2020; Liu et al., 2020f; Nissen et al., 2020; van Doremalen et al., 2020; Zhang and Duchaine, 2020; Alsved et al., 2022), with an estimation of about 4.8×10^5^ virus gene copies/mL in bioaerosol samples (Cevik et al., 2021; Malik et al., 2021; Johnson et al., 2022).

On the other hand, we must consider that with SARS-CoV-2, it has been shown that contagiousness commonly occurs before developing any symptom (Arons et al., 2020; Zhou et al., 2020b; Le et al., 2020; Mirjalali et al., 2020; Moghadas et al., 2020; Bae et al., 2021; Muller, 2021; Murata et al., 2021; Wilmes et al., 2021; Fakhruddin et al., 2022; Mugglestone et al., 2022; Overton et al., 2022; Pettifor et al., 2022; Rajme-Lopez et al., 2022), compared with seasonal flu, for example, where most transmissions occur after a person has developed symptoms (Leung et al., 2015b; Ip et al., 2017; Furuya-Kanamori and Yakob, 2018). The average SARS-CoV-2 incubation period is thought to be about 5 days, while peak infectiousness begins 2 days before symptoms reveal themselves (Yan et al., 2020b; Zhou et al., 2020b; Hua et al., 2020; McAloon et al., 2020; Noh et al., 2020; Tan et al., 2020; Bae et al., 2021; Murata et al., 2021; Yan et al., 2021). Moreover, viral loads in asymptomatic and symptomatic infected individuals have been reported to be similar (Arons et al., 2020; Lee et al., 2020b; Zhou et al., 2020b; Hoehl et al., 2020; Kimball et al., 2020; Kociolek et al., 2020; Le et al., 2020; Sohn et al., 2020; Uhm et al., 2020; Zou et al., 2020; Hasanoglu et al., 2021; Ra et al., 2021; Sah et al., 2021; Zuin et al., 2021), as the efficiency to transmit the virus (Li et al., 2020a; Arons et al., 2020; Wong et al., 2020a; Bae et al., 2020; Gao et al., 2020b; Kim et al., 2020b; Li et al., 2020b; Qian et al., 2020b; Zhou et al., 2020b; Day, 2020; Wang et al., 2020g; Hao et al., 2020; He et al., 2020; Jiang et al., 2020; Moghadas et al., 2020; Rivett et al., 2020; Sohn et al., 2020; Glenet et al., 2021; Hasanoglu et al., 2021; Joshi et al., 2021; Passarelli et al., 2021; Sah et al., 2021). Hence, the majority of infections occur pre-symptomatically, with the infected people ignoring that they have the disease (Ferretti et al., 2020; He et al., 2020; Moghadas et al., 2020).

**FIGURE 2:**
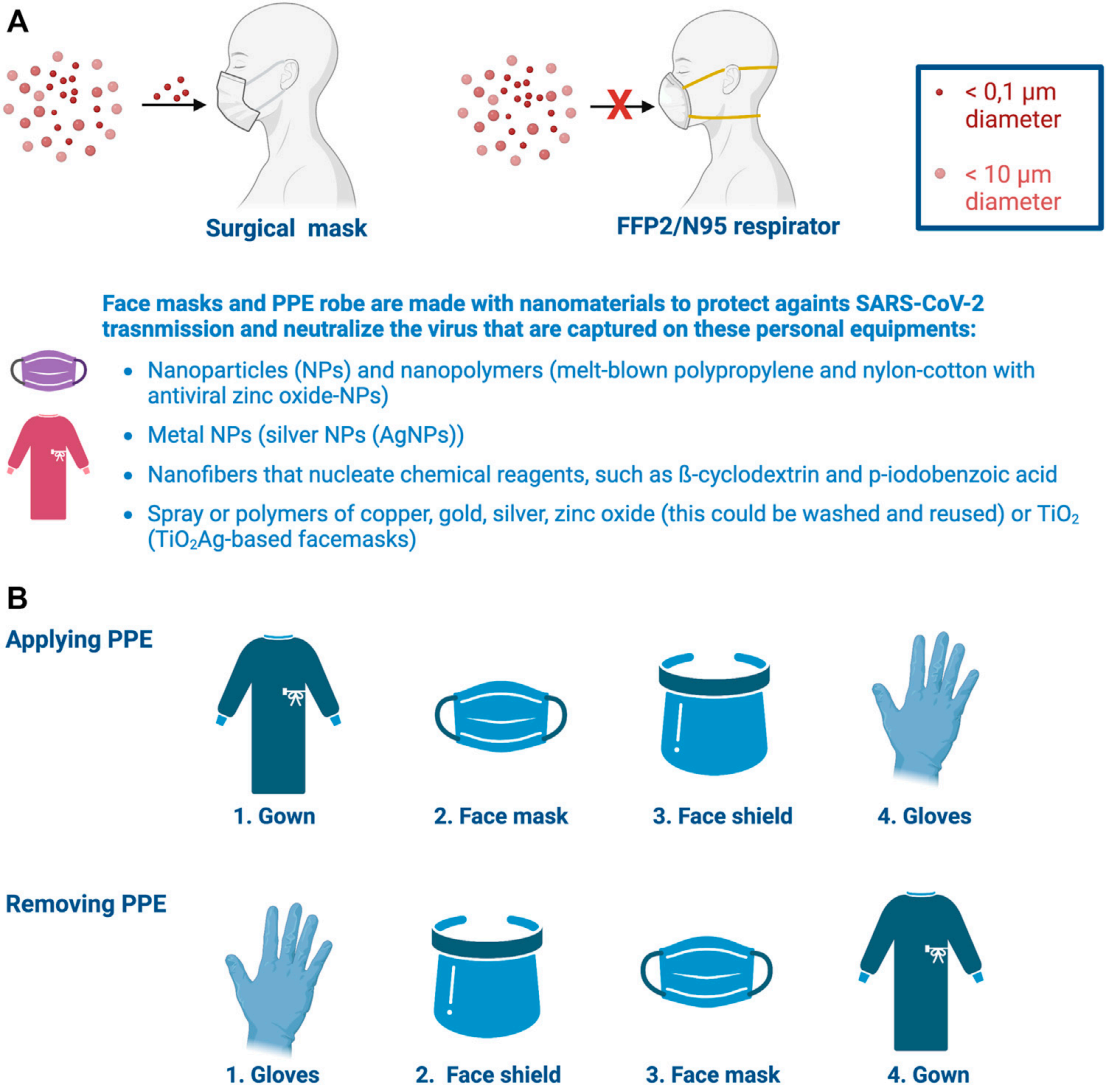
Nanomaterials used for protective personal equipment. **(A)** FFP2/NP95 masks are the appropriate protective respirators against this virus mainly transmitted by aerosol compared with surgical masks. Face masks and PPE robes have been incorporated nanoreagents and nanomaterials to improve personal protection against SARS-CoV-2 airborne infection and transmission to neutralize the virus in contact with these personal equipments. These nanomaterials are indicated in panel **(A)**. **(B)** Personal training and correct protocols for wearing (applying) and removing these PPEs from the body are critical to ensure right protection of health personnel against SARS-CoV-2 infection. Designs and templates are created with BioRender.

In this pandemic scenario, the most basic method of prevention against COVID-19 is therefore to wear face masks (Howard et al., 2021; Ju et al., 2021). The face mask must be worn by either non-infected or infected people (Figure 2A). Thus, in infected individuals, facemasks could prevent the aerosol spread of the virus to other people, whereas the mask could protect against SARS-CoV-2 in non-infected individuals by covering their faces (Pezzini and Padovani, 2020; Catching et al., 2021). Face masks retain on their surface of the textile the virus and the constant use of this PPE (protective personal equipment) forces to renew the mask constantly, particularly in people who are exposed to the virus as in shops, pharmacies, schools, administration buildings, offices and hospitals, among other professions and sensitive infrastructures to reduce viral transmission and infection (Beesoon et al., 2020; Sanchez et al., 2020; Forum, 2020; Gholami et al., 2021; Howard et al., 2021; Ingram et al., 2021; Lessler et al., 2021). N95 and filtering facepiece 2 and 3 (FFP2 and FFP3) respirators that can filter from 95 to 99% of airborne particles, respectively, and surgical masks (filters around 80% of contaminants of certain size) are the most common face masks used during COVID-19 pandemics which provide protection against the airborne virus and larger infectious particles (Bartoszko et al., 2020; Karim et al., 2020; Sommerstein et al., 2020; Clinkard et al., 2021; Dheda et al., 2021; Regli et al., 2021; Rohit et al., 2021) (Figure 2A). These face masks have also been improved by using nanomaterials that present antimicrobial and antiviral properties, such as nanoparticles (NPs) (i.e., melt-blown polypropylene and nylon-cotton with antiviral zinc oxide-NPs) (Gonzalez et al., 2021), metal NPs (i.e., silver NPs (AgNPs)) (Valdez-Salas et al., 2021), spray or polymers of copper, gold, silver, zinc oxide (this could be washed and reused) or TiO_2_ (TiO_2_Ag-based facemasks are able to degrade 99.99% of viruses under zero light conditions) (Balagna et al., 2020; Konda et al., 2020; Mahapatra et al., 2020; Nickels, 2020; Sportelli et al., 2020; Wang et al., 2021a; Nbic+, 2022; Promethean-Particles-Ltd, 2022; Sonovia-SonoMask, 2022), in order to stop the virus transmission chain (Figure 2A). These metal oxide-derived NPs, embedded in face masks, act as antiviral or, in general, as antimicrobial compounds by inhibiting the catalytic activity of the microorganisms’ enzymes (i.e, viral polymerases and proteinases), which are key for their survival and pathogenicity (Cai et al., 2019; Wang et al., 2021a; Melk et al., 2021; Pachaiappan et al., 2021; Mendes et al., 2022). Moreover, due to their small size, these metal oxide-derived NPs enhance the surface contact with viruses and bacteria, as reported for Ag-TiO_2_ single atom nanozyme (SAN) that allows efficient adsorption for SARS-CoV2 through the association of Ag atoms with Cys and Asn residues of the RBD sequence of the viral S1 subunit (Wang et al., 2021a). These metal oxide-derived NPs, in contact with virus could also trigger the formation of reactive oxygen species (i.e., hydrogen peroxide anions and superoxide radical anions) which cause damage to the viral membranes and cell wall of bacteria, as well as to internal components, thereby impairing their uncoating, growth and integrity (Cai et al., 2019; Hamza et al., 2021; Melk et al., 2021; Pachaiappan et al., 2021; Mendes et al., 2022). Noteworthy, the effects of these metal oxide-derived NPs could depend on their composition and dose (Zanet et al., 2019; Wang et al., 2021a). Therefore, the use of these NPs, polymers and sprays for face masks production or treatment implies accurate techniques and procedures, in order to assure the maximal protection of face masks against SARS-CoV-2.

All these nanoparticles are also used to produce PPE robe (Reddy et al., 2019; Bauchner et al., 2020; JH et al., 2020; Ortega et al., 2020) (Figure 2B), where PPE innovation will also help to overcome the important COVID-19 PPE waste challenge (IFC, 2021). Face masks and PPE robes have been also improved to fight and prevent COVID-19/SARS-CoV-2 infection by using nanofibers to create a dense weblike surface area against infection in the PPEs (Figure 2). These webs of nanofibers could nucleate some chemical reagents, such as β-cyclodextrin and p-iodobenzoic acid that allow nanofibers to act against SARS-CoV-2, thereby degrading the virus and reducing the risk of inhaling the virus or to be contaminated by contact with masks or PPE robes carrying the virus (Ramaseshan et al., 2006; Kadam et al., 2021; Rasmi et al., 2021; Owida et al., 2022). The nanofibers could be also presented in a variety of layers, including a water repellent and a skin-friendly silk layer with a 98% filtration guide, leading to an efficient protective PPE mask or robe that avoid SARS-CoV-2 transmission and infection (Chaudhary et al., 2021). Moreover, it has been reported that SARS-CoV-2 can remain on fomites for more than 9 days and potentially infectious (Kampf et al., 2020b; Bueckert et al., 2020; Hosseini et al., 2021; Marcenac et al., 2021). Therefore, several nanomaterials have been used to disinfect or remove this Sarbecovirus from different contaminated surfaces, such natural NPs-, metal NPs- and nanopolymers-based disinfectants (reviewed in (Souri et al., 2022)).

### 2.2 COVID-19 vaccines by nano-based strategies: Prevention and treatment

Physical barriers are required and are therefore very useful to protect ourselves and the others from infection, contagion and community spreading of the virus, lowering the concomitant emergence of viral variants (Střížová et al., 2020; Swain, 2020; Mello et al., 2022), which threatens individual and community immunity that might have been generated during previous infections or by vaccines (Arora et al., 2021; Boehm et al., 2021; Caniels et al., 2021; Arora et al., 2022; Salehi-Vaziri et al., 2022), as it has happened with the appearance of several Omicron VOCs (Cao et al., 2022; Flemming, 2022; Hachmann et al., 2022). Therefore, vaccines are the tools to elicit immunity against SARS-CoV-2 infection, reinfection by VOCs and severe COVID-19 disease, where face masks enhance vaccine efficiency in this crucial community and worldwide task. The community use of face masks therefore lower both virus transmission rates and the severity of the disease allowing to save more lives (Klompas et al., 2020b; Esposito et al., 2020; Gandhi and Rutherford, 2020; Leung et al., 2020). Likewise, it should be considered that combining vaccination and face masks is much less costly to apply than the use of a plethora of non-efficient drugs together with hospital intensive cares measures which are associated with high economic, social and live costs (Beesoon et al., 2020; Esposito et al., 2020; George et al., 2020; Grasselli et al., 2020; Hill et al., 2020; Iannaccone et al., 2020; Lerner et al., 2020; Rosenthal et al., 2020; Shaffer, 2020; Singh et al., 2020; Cleary et al., 2021; Di Fusco et al., 2021; Kompaniyets et al., 2021; Ohsfeldt et al., 2021; Bartsch et al., 2022; Brüssow and Zuber, 2022; Popescu et al., 2022).

Before presenting the different vaccine designs used against SARS-CoV-2/COVID-19, we summarize the immune responses elicited against SARS-CoV-2 infection and how the virus pervert immune protection leading to the COVID-19 pathology.

The innate immune responses are rapidly developed against this Sarbecovirus which very early during the infection leads to the production of type I interferons (IFNs), key antiviral cytokines that induce a large range of proteins that impair viral replication in infected cells (Lokugamage et al., 2020; Madden and Diamond, 2022). SARS-CoV-2 evades the host innate immune system by reducing IFN levels, its mediated signals and activation pathways through different SARS-CoV-2 proteins (Shin et al., 2020a; Lei et al., 2020; Miorin et al., 2020; Xia et al., 2020; Wang et al., 2021c; Wang et al., 2021d; Hayn et al., 2021; Kimura et al., 2021; Min et al., 2021; Munnur et al., 2021; Rashid et al., 2021; Vazquez et al., 2021; Chiale et al., 2022; Oh and Shin, 2022; Osipiuk et al., 2022; Znaidia et al., 2022). Moreover, genetic and immunological defects in the type-I IFN responses are known to cause fatal outcomes in otherwise healthy SARS-CoV-2 infected patients (Zhang et al., 2020d; Zhang et al., 2022a). Likewise, SARS-CoV-2 escape the translation inhibition as a result of some viral proteins (i.e., Nsp14 with the concert of Nsp10) (Hsu et al., 2021) and mRNAs that elude translation inhibition by a stem loop structure in the 5ʹ untranslated region (UTR) of all viral transcripts (Tidu et al., 2020).

Adaptative immune responses against SARS-CoV-2 antigens are important to control viral infection and the severity of the COVID-19 disease (Rydyznski Moderbacher et al., 2020). Specific and effective CD8^+^ T cell response is associated with viral clearance and mild disease (Bergamaschi et al., 2021; Notarbartolo et al., 2021). In acute COVID-19, effective CD8^+^ and CD4^+^ T cell responses are associated with positive clinical outcomes (Diao et al., 2020; Su et al., 2020; Notarbartolo et al., 2021; Rha et al., 2021). This T cell activation is also observed in asymptomatic SARS-CoV-2 infected individuals (Grau-Exposito et al., 2021; Le Bert et al., 2021). However, exacerbated T cell responses could exhaust these immune cells, being associated with poor clinical outcomes (Lucas et al., 2020b; Diao et al., 2020; Mathew et al., 2020; Song et al., 2020; Su et al., 2020), particularly in patients presenting comorbidities (Lucas et al., 2020b; Yu et al., 2021). In fact, humoral response has been reported to be retarded in patients with severe COVID-19 and in fatal outcomes (Lucas et al., 2021). Follicular T and B cell cooperation in germinal centers (GCs) are key to create specific memory cells against viral antigens and humoral and cellular long-lasting repertoires and responses (Allen et al., 2007; Kräutler et al., 2017; Cyster and Allen, 2019). Severe SARS-CoV-2 infection and illness have been associated with the absence of GCs (Kaneko et al., 2020) as observed by the decrease in the number of T follicular cells. This could be in agreement with the robust extrafollicular B cell responses with concomitant increase in proinflammatory cytokine levels and the neutralizing antibody titers observed in severe COVID-19 patients with critical pneumonia (Woodruff et al., 2020), where delayed production and responses of specific neutralizing antibodies (nAbs) (Lucas et al., 2021) and the pre-existence of type-I IFN auto-Abs (Bastard et al., 2020; Bastard et al., 2021; Wang et al., 2021b) have been reported.

**FIGURE 3:**
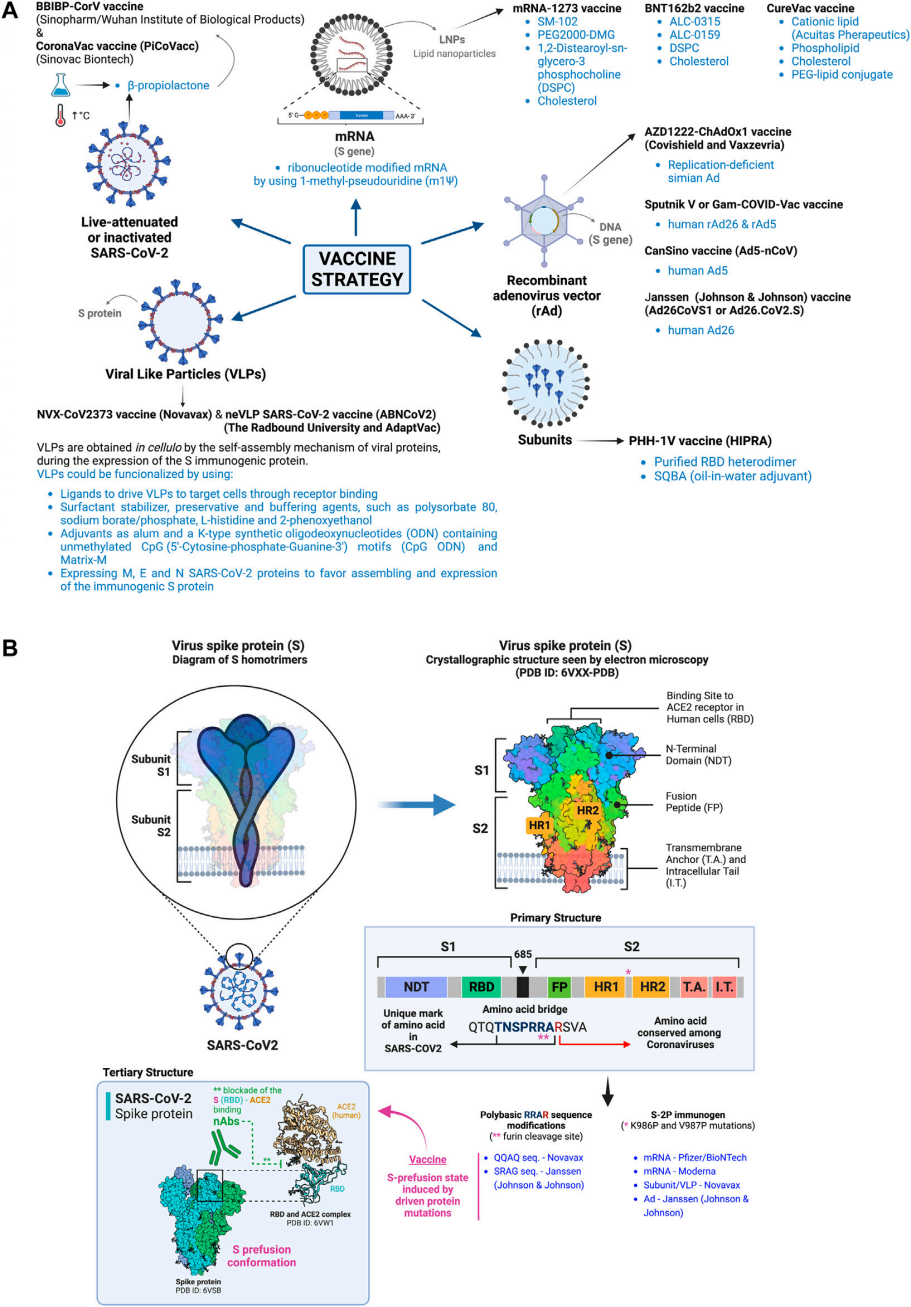
Nano-based COVID-19 vaccines. **(A)** The scheme summarizes the different nano-based strategies used for immunization against SARS-CoV-2 infection and severe COVID-19 based or different nanomaterials and reagents to deliver the modified viral genome (coding for the immunogen S protein) and recombinant immunogen S protein or its subunits, such as the LNP-mRNA vaccines, the rAd vectors containing the DNA S gene, chemically inactivated SARS-CoV-2 viral particles, VLPs bearing recombinant S protein, and recombinant subunit vaccines (i.e., adapted full-length S protein or fragments as the RBD region) in emulsion with nanoreagents. All nanomaterials and nanotechnology used for vaccine development are indicated in the different vaccine strategies. **(B)** Schemes represent the primary and tertiary structures of the S viral protein, used as immunogen for vaccine developments, stabilized in its prefusion state by two key protein mutations (K986P and V987P; named S-2P immunogen) and modifying the polybasic domain (RRAR) to render it non-cleavable by the furin proteinase. The S protein domains and its trimeric organization on virus membrane and on infected-cell plasma membrane are shown, together with the RBD sequence where nAbs bind to neutralize SARS-CoV-2/ACE2 interaction and subsequent virus infection. The S-associated modifications introduced by the different vaccine designs are indicated. Designs and templates are created with BioRender.

Therefore, effective vaccine designs against SARS-CoV-2 infection and COVID-19 disease should induce robust and persistent GC reactions, in order to generate high-affinity and durable neutralizing antibody responses together with T cell memory coordinated responses and reservoir (Brouwer et al., 2020; Jagannathan and Wang, 2021; Teijaro and Farber, 2021; Turner et al., 2021; Winkler et al., 2021). Innovative nanovaccine formulations, as we further describe, have demonstrated to successfully achieve optimal activation of both innate and adaptive immune responses against SARS-CoV-2 infection (i.e., mRNA vaccines-mediated GC-B cell response to SARS-CoV-2 (Lederer et al., 2020; Turner et al., 2021; Laidlaw and Ellebedy, 2022)), in order to achieve herd immunity (reviewed in (Shin et al., 2020b; Chauhan et al., 2020; Chung et al., 2020; Constantin et al., 2021; Machhi et al., 2021; Guerrini et al., 2022)). Apart from the efficacy and immunogenicity of vaccines, safety and protection against reinfections are other challenges that must be considered in the development of vaccines (WHO, 2020; Chen et al., 2021b; Liu et al., 2021c; Hasanzadeh et al., 2021; Teijaro and Farber, 2021). The spike (S) gene/protein of SARS-CoV-2 has been chosen as the main antigen to design vaccines to immunize, either by itself (i.e., in form of mRNA or derived DNA gene or recombinant protein) or present as protein in attenuated or inactive viral particles or in viral like particles (VLPs) (Heinz and Stiasny, 2021; Martinez-Flores et al., 2021) (Figure 3A), because the targeting of this protein with vaccine-elicited nAbs could be important to impair the entry of the virus into the host cells and cause infection (Figure 3B, *see inbox: nAbs action in the S-tertiary structure*), therefore, inhibiting viral replication, spread and cause the disease (Brouwer et al., 2020; Corbett et al., 2021; Earle et al., 2021; Khoury et al., 2021; McMahan et al., 2021) (see Figure 1A,B).

The design and generation of the above indicated genomic or protein-based viral immunogens are part of the nanotechnology employed for vaccination (Chauhan et al., 2020; Wang et al., 2020d), summarized in Figure 3. Moreover, nano-based technology used for vaccine formulation and immunogen administration has been useful to achieve specific anti-viral immune responses and facilitate the positive outcome from severe SARS-CoV-2 infection and illness (Kräutler et al., 2017; Anderson et al., 2020; Walsh et al., 2020a; Walsh et al., 2020b; Chung et al., 2020; Jackson et al., 2020; Mulligan et al., 2020; Polack et al., 2020; Heath et al., 2021; Meo et al., 2021; Turner et al., 2021; Vahedifard and Chakravarthy, 2021; Widge et al., 2021; Toback et al., 2022). Indeed, the use of these NPs or reagents in vaccine designs protect the genomic o protein immunogen from fast degradation by host proteinases, thus enhancing their stability (Miles et al., 2005; Tinkle et al., 2014; Zhao et al., 2014; Chung et al., 2020). Likewise, NPs facilitate the delivery of the vaccine in the organism and the further uptake of viral antigens by antigen-presenting cells (APCs) (Schwendener, 2014; Tinkle et al., 2014; Zhu et al., 2014; Chung et al., 2020). Vaccine stability and solubility has been promoted by the use of NPs such as liposomes (Peek et al., 2008; Alving et al., 2016; Bulbake et al., 2017; Perrie et al., 2017), lipids (Sivakumar et al., 2011; Baz Morelli et al., 2012; Comber and Philip, 2014; Schwendener, 2014; Cibulski et al., 2016), biodegradable polymers (i.e., polyanhydride copolymers based on 1,8-bis-(p-carboxyphenoxy)-3,6-dioxaoctane (CPTEG), 1,6-bis-(p-carboxyphenoxy)-hexane (CPH), and sebacic anhydride (SA)) or dendrimer-based formulations (Chahal et al., 2016; Wafa et al., 2017; Banerjee et al., 2019; Liu et al., 2021b) and emulsions (i.e., MF59^®^, squalene and Montanide ISA™51) (Miles et al., 2005; Ascarateil and Dupuis, 2006; Della Cioppa et al., 2012; O'Hagan et al., 2013; Della Cioppa et al., 2014; Ascarateil et al., 2015; Shah et al., 2015; van Doorn et al., 2016; Kantipakala et al., 2019; Savoji et al., 2019). The nature or the physicochemical characteristics of these NPs determine the efficiency of antigen delivery and the potential adverse effects observed during immunization (reviewed in (Mitchell et al., 2021)). Thus, lipid-based NPs (LNPs), such as liposomes and emulsions, allow simplicity during NPs formulation and self-assembly, offering high biocompatibility, bioavailability, and flexibility to accommodate different antigen sizes (Fonseca-Santos et al., 2015; Sercombe et al., 2015), since LNPs could be modified for these purposes during synthesis (Alyautdin et al., 2014; Leung et al., 2015a; Sercombe et al., 2015; Sarfraz et al., 2018; Sedighi et al., 2019). Moreover, LNPs allow to deliver hydrophilic, hydrophobic or lipophilic agents, as well as lipophilic and hydrophilic compounds together in the same LNP preparation (Alyautdin et al., 2014; Fonseca-Santos et al., 2015; Sercombe et al., 2015; Sedighi et al., 2019). Although LNPs achieve low encapsulation efficiency, they are the most approved NP system for drug delivery (Fenton et al., 2018; Anselmo and Mitragotri, 2019), as occurred with the mRNA-based vaccines against SARS-CoV-2 infection and COVID-19 disease (reviewed in (Tenchov et al., 2021)). Natural (i.e., heparin, hyaluronan, chitosan or dextran (Lombardo et al., 2019; Zielińska et al., 2020)) or synthetic [i.e., saturated poly (α-hydroxy esters), such as poly (lactic acid) (PLA), poly (glycolic acid) (PGA), and poly (lacticcoglycolide) (PLGA) (Shive and Anderson, 1997; Lin et al., 2012; Chenthamara et al., 2019; Gangapurwala et al., 2020; Zielińska et al., 2020)] polymeric NPs, such as dendrimers or nanospheres, permit the precise control of the physicochemical particle characteristics, allowing posterior surface modifications and being flexible for hydrophilic and hydrophobic cargo accommodation (Kannan et al., 2014; Xu et al., 2014; Patra et al., 2018; Zhang et al., 2020b; Pinelli et al., 2020; Valcourt et al., 2020; Volpatti et al., 2020; Zielińska et al., 2020). These characteristics together with their biocompatibility, biodegradability and low toxicity (Chenthamara et al., 2019; Lombardo et al., 2019; Pinelli et al., 2020) make polymeric NPs a good choice for vaccine delivery (Zhang et al., 2020b). Of note, these types of polymeric NPs could present a high degree of aggregation that could be toxic and difficult antigen delivery, therefore compromising the optimal antigen concentration in target cells and tissues for immunization (Knight et al., 2019; Zielińska et al., 2020).

These NPs or vehicles also favor immunogenicity because they act as adjuvants triggering the immune cell activation and promoting immune responses (Ascarateil and Dupuis, 2006; Sivakumar et al., 2011; Baz Morelli et al., 2012; Zhu et al., 2014; Ascarateil et al., 2015; Varypataki et al., 2015; Alving et al., 2016; Cibulski et al., 2016; Khabazzadeh Tehrani et al., 2016; Perrie et al., 2017; Wafa et al., 2017; Khademi et al., 2018; Kantipakala et al., 2019; Savoji et al., 2019; Petkar et al., 2021). The NP surface could be modified by presenting monosaccharides (i.e., mannose) or immune ligands (i.e., toll-like receptor ligands), in order to improve elicited vaccine delivery of the immunogen and immune stimulation (Apostolopoulos et al., 2013).

In this scenario against SARS-CoV-2/COVID-19, LNPs have been developed and used to deliver and immunize with modified mRNA of the entire S protein or only the RBD segment of the viral glycoprotein (i.e., novel LNP-encapsulated mRNA based vaccine (Wang et al., 2020a) or LNP-encapsulated mRNA encoding RBD vaccine (Tai et al., 2020)) (Figure 3A), or by using a self-amplifying RNA (saRNA or SAM) (Perri et al., 2003) that encodes the alphaviral replicase to drive the expression of a pre-fusion stabilized SARS-CoV-2 S-gene/protein. This strategy allows to reduce the injection dose (tenfold lower than mRNA vaccines) and follow a “one-dose prime-boost” regime (Vogel et al., 2018; Erasmus et al., 2020; Schoenmaker et al., 2021) instead of the “two-dose” strategy per recipient of the non-replicative mRNA-COVID-19 vaccines. SAM vaccines in development are the LNP-nCoV-saRNA vaccine by the Imperial College London (McKay et al., 2020; Schoenmaker et al., 2021), and the ARCT-021 or LUNAR-COV19 vaccine (see information in https://www.nature.com/articles/d43747-021-00073-3) by Arcturus/Duke-NUS (Low et al., 2021; Schoenmaker et al., 2021). The LNP-nCoV-saRNA vaccine has been assayed in a Phase I clinical trial, being immunogenic at low dose levels, but failed to induce 100% seroconversion. Some modifications are being developed to optimize humoral responses against the viral S antigen (Pollock et al., 2022). In the case of ARCT-021 (LUNAR-COV19) vaccine, the mRNA is a replicon that comprises the Venezuelan equine encephalitis virus (VEEV) genome in which the structural genes have been replaced with the SARS-CoV-2 full-length S-gene, and formulated with the proprietary LUNAR^®^ LNP (Low et al., 2021). ARCT-021 protects K-18 human ACE2 transgenic mice from lethal SARS-CoV-2 challenge producing a strong Th1-predominant humoral and cellular immune responses against the S protein (de Alwis et al., 2021), with promising 1–2 phase clinical trial results (Low et al., 2021).

In the case of liposomes, Moderna’s candidate vaccine mRNA-1273 (Moderna/National Institute of Allergy and Infectious Diseases; commercially available under the brand name Spikevax) for COVID-19 used them as a carrier for drug delivering and as an adjuvant, thereby encapsulating the nucleoside modified mRNA that encodes the SARS-CoV-2 S glycoprotein (Callaway, 2020; Huang et al., 2020c; Baden et al., 2021; Dai and Gao, 2021; Tenchov et al., 2021), whereas LNPs were used for this purpose by BioNTech/Fosun Pharma/Pfizer, BNT162b2 mRNA vaccine (commercially available under the brand name Comirnaty) (Callaway, 2020; Polack et al., 2020; Baden et al., 2021; Dai and Gao, 2021; Tenchov et al., 2021) (Figure 3A). The LNPs components of these two efficient mRNA vaccines are: 1) for mRNA-1273, SM-102, PEG2000-DMG, 1,2-Distearoyl-sn-glycero-3 phosphocholine (DSPC) and cholesterol; 2) for BNT162b2, ALC-0315, ALC-0159, DSPC and cholesterol (reviewed in (Kim et al., 2021a; Schoenmaker et al., 2021; Verbeke et al., 2021)) (Figure 3A). Furthermore, Pfizer/BioNTech and Moderna vaccines both uses a ribonucleotide modified mRNA by using 1-methyl-pseudouridine (m1Ψ) instead of uridine (Polack et al., 2020; Baden et al., 2021) (Figure 3A). In fact, the m1Ψ nucleotide is a natural archaeal tRNA component (Wurm et al., 2012) that could be chemically synthesized and used for production of m1Ψ mRNAs by *in vitro* transcription (reviewed in (Nance and Meier, 2021)). The presence of the m1Ψ nucleotide in the mRNA construct provokes changes in the secondary structure of the mRNA that has been associated with high protein expression (Andries et al., 2015; Zhao and He, 2015; Svitkin et al., 2017; Van der Jeught et al., 2018; Foster et al., 2019; Hadas et al., 2019; Mauger et al., 2019; Nelson et al., 2020; Parr et al., 2020; Nance and Meier, 2021; Mokuda et al., 2022). Likewise, the introduction of these m1Ψ nucleotides inside mRNA fragments has been reported to stabilize the mRNA and reduce its recognition by RNA-binding proteins, thereby minimizing undesirable innate immune responses against this foreign mRNA immunogen (Andries et al., 2015; Zhao and He, 2015; Van der Jeught et al., 2018; Foster et al., 2019; Mauger et al., 2019; Nelson et al., 2020; Nance and Meier, 2021; Mokuda et al., 2022).

Moreover, Pfizer/BioNTech and Moderna vaccines both uses a mRNA encoding a modified S protein stabilized in its prefusion conformation (Erasmus et al., 2020; Hsieh et al., 2020; Wrapp et al., 2020), in order to maintain a stable conformational state exposing the S1 distal domain of the S protein, against which vaccine-elicited protective nAbs are aimed to be generated (Erasmus et al., 2020; Hsieh et al., 2020; Xia, 2021) (Figure 3B, *see inbox: nAbs in the S-tertiary structure*). The strategy to stabilize the immunogenic prefusion conformation on the membrane-anchored S protein consists in modifying two key amino acid residues for proline in the S protein (i.e. K986P and V987P), leading to the known S-2P immunogen (Walsh et al., 2020b; Corbett et al., 2020; Olia et al., 2021; Xia, 2021) (Figure 3B). These consecutive residues are located in the central helix region of the S protein, a segment placed between the Heptad-Repeat (HR) domains HR1 (912–984 residues) and HR2 (1,163–1,213 residues) of the S protein (Huang et al., 2020c; Hsieh et al., 2020; Xia, 2021) (Figure 3B). The K986P and V987P mutations prevent structural transitions from prefusion to postfusion state, thereby stabilizing the S-2P protein at the prefusion state and contributing to vaccine efficiency (Huang et al., 2020c; Hsieh et al., 2020; Xia, 2021) (Figure 3B). Other vaccine developments that contain the S-2P immunogen are the Sanofi design, the subunit/VLP Novavax vaccine and the Janssen (Johnson and Johnson) adenovirus (Ad)-based vaccine (Guebre-Xabier et al., 2020; Mercado et al., 2020; Goepfert et al., 2021). Furthermore, another strategy to stabilize the immunogenic prefusion conformation of the SARS-CoV-2 S glycoprotein consists in changing the furin cleavage site in the S protein (Andersen et al., 2020), in order to avoid this cleavage that releases the S1 subunit of the S viral protein that would compromise the appropriate immunogenic state of the S protein for vaccination (Walls et al., 2020b; Wrapp et al., 2020; Johnson et al., 2021; Olia et al., 2021). In fact, the SARS-CoV-2 S protein must be cleaved into the S1 and S2 subunits, in order to be able to trigger fusion and host-cell entry (Hoffmann et al., 2020a; Walls et al., 2020b; Jaimes et al., 2020) (Figure 1B). SARS-CoV-2 presents a four-amino acid “SPRRAR|S” sequence (preceded with P) named as the polybasic site or the furin cleavage site, which is inserted at the junction of the S1 (containing the RBD sequence) and S2 (bearing the fusion domain) subunits of the S glycoprotein (Andersen et al., 2020) (Figure 3B). This polybasic site has been associated with the pathogenesis of SARS-CoV-2 infection and COVID-19 pandemic severity (Hoffmann et al., 2020a; Andersen et al., 2020; Shang et al., 2020b; Walls et al., 2020b; Jaimes et al., 2020; Johnson et al., 2020; Lau et al., 2020; Lemmin et al., 2020; Johnson et al., 2021; Whittaker, 2021; Villoutreix et al., 2022). It is interesting to note that the sequence of the ancestral (Wuhan-Hu-1) SARS-CoV-2 furin cleavage site (RRxR) does not follow those of the prototypical furin cleavage sites (RxK/RR) (Tang et al., 2021), and that during the COVID-19 pandemic this site has been adapted in some VOCs, such as B.1.1.7 (Alpha) and B.1.617 (B.1.617.1 named Kappa and B.1.617.2 named Delta) (Aleem et al., 2022) where the first proline has been replaced by a histidine (HRRAR|S) or an arginine (RRRAR|S), respectively (Peacock et al., 2021b; Neerukonda et al., 2021; Whittaker, 2021; Lubinski et al., 2022). Altogether, this information shows that some vaccine designs have incorporated mutations at the furin cleavage site to improve the immunogenic activity of the S-2P immunogen (Figure 3B). Furthermore, the Ad26CoVS1 or Ad26. CoV2 vaccine [Janssen Pharmaceutical Companies (Johnson and Johnson)] presents a substitution of the S-2P “RRAR (R682-R685)” sequence with the non-cleavable SRAG sequence (Bos et al., 2020; Mercado et al., 2020; Dai and Gao, 2021), whereas the NVX-CoV23 vaccine (Novavax) substitutes the furin-polybasic domain (R682-R685) with the non-cleavable QQAQ sequence (Bangaru et al., 2020; Keech et al., 2020; Dai and Gao, 2021; Tian et al., 2021) (Figure 3B).

Noteworthy, other mRNA-based LNP-driven strategies have also been developed, such as the CureVac N.V. vaccine (i.e., CVnCoV vaccine containing sequence-optimized mRNA coding for a stabilized form of SARS-CoV-2 S protein encapsulated in LNP (Kremsner et al., 2021; Rauch et al., 2021; Sáez-Llorens et al., 2022) (Figure 3A), which was expected to be cheaper and more stable in refrigerated storage than those developed by Pfizer-BioNTech and Moderna. These last vaccines contain a pseudouridine mRNA nucleotide in place of the normal uridine nucleotide to circumvent immune inflammatory reactions (Karikó et al., 2005) that could be activated through detection of the foreign S viral mRNA (Morais et al., 2021) (Figure 3A). Although CureVac’s vaccine bears normal uridine (Kremsner et al., 2021), it was developed altering the S viral mRNA genome code in a way that the protein is not affected (Kremsner et al., 2021), but helps to evade immune detection ((Rauch et al., 2021; Sáez-Llorens et al., 2022); analyzed in (Dolgin, 2021)). The formulation of CurveVac’s LNPs consists in cationic lipid (Acuitas Therapeutics), phospholipid, cholesterol and PEG-lipid conjugate (reviewed in (Kim et al., 2021a; Schoenmaker et al., 2021; Verbeke et al., 2021)) (Figure 3A). Unfortunately, CureVac’s mRNA vaccine falls on efficacy in clinical trials (analyzed in (Baker and Dolgin, 2021; Dolgin, 2021)). All the nanocarriers used in the above vaccines protect the immunogen against biodegradation and favor their uptake inside cells that should express and present the immunogen to the immune system (Apostolopoulos et al., 2013; Callaway, 2020; Asdaq et al., 2021; Souri et al., 2022).

Another nano-strategy followed to develop a vaccine against COVID-19 is the use of viral vector vaccines to deliver the S gene and express the S protein of SARS-CoV-2 to trigger specific and protective immune responses (Callaway, 2020; Premkumar et al., 2020; Dai and Gao, 2021), such as well-known adenoviruses (Ads) (Tatsis and Ertl, 2004) (Figure 3A). Hence, recombinant non-replicating Ad (rAd) has been used by the University of Oxford/AstraZeneca (AZD1222-ChAdOx1 vaccine; a replication-deficient simian Ad-vectored vaccine) (commercially available under the brand names Covishield and Vaxzevria), Gamaleya National Research Centre for Epidemiology and Microbiology (Sputnik V or Gam-COVID-Vac; rAd26 and rAd5 vaccines), CanSino Biological Inc./Beijing Institute of Biotechnology (Ad5-nCoV vaccine) and Janssen Pharmaceutical Companies (Johnson and Johnson) (Ad26CoVS1 or Ad26.CoV2.S vaccine) (Zhu et al., 2020a; Bos et al., 2020; Zhu et al., 2020b; Callaway, 2020; Folegatti et al., 2020; Logunov et al., 2020; Mercado et al., 2020; Sadoff et al., 2021a; Sadoff et al., 2021b; Creech et al., 2021; Ewer et al., 2021; Logunov et al., 2021; Ramasamy et al., 2021; Stephenson et al., 2021; Swanson et al., 2021; Voysey et al., 2021) (Figure 3A).

The use of the attenuated or inactivated natural SARS-CoV-2 virus is another strategy that has been applied in this important COVID-19 pandemic crisis (Gao et al., 2020a; Wang et al., 2020b; Xia et al., 2021), due to the potential efficacy and production speed (Figure 3A). This procedure implies the culturing of high amounts of infectious viral strains, isolated from patients, expanded, for example, in infected Vero cells as performed for the BBIBP-CorV (Sinopharm/Wuhan Institute of Biological Products) and CoronaVac vaccine (formerly named PiCoVacc; Sinovac Biontech) vaccines (Gao et al., 2020a; Wang et al., 2020b) (Figure 3A). The Vero continuous cell line (CCL) allows highly efficient virus proliferation and conserves its genetic stability, being both requirements for the development of an inactivated virus vaccine. Hence, high viral genome homology between the expanded virus and the primary strain must be confirmed, as it was analyzed for BBIBP-CorV vaccine with no amino acid variations observed within ten passages (Wang et al., 2020b) or for PiCoVacc vaccine (Gao et al., 2020a). In fact, Vero was the first CCL approved by the WHO to produce human vaccines (WHO, 1987; Barrett et al., 2009; Kiesslich and Kamen, 2020). Thus, amplified and purified infectious viral particles are further inactivated by different procedures. Here, we would like to remark the viral chemical inactivation as performed for the BBIBP-CorV vaccine (Wang et al., 2020b). This is inactivated by using β-propiolactone (at a ratio of 1:4,000 at 2–8°C for 20–24 h) (Figure 3A). The BBIBP-CorV vaccine also contains aluminum hydroxide (alum) as the adjuvant (Wang et al., 2020b) and it has been reported as safe, well tolerated inducing high humoral responses (Xia et al., 2021; Xia et al., 2022). The Sinopharm/Wuhan Institute of Biological Products consortium has also developed two other inactivated virus vaccines based on two primary viral strains, WIV04 and HB02 (Al Kaabi et al., 2021) which offer 72.8 and 78.1% of protection, respectively, against moderate-to-severe forms of the COVID-19 disease. The Sinovac Biontech virus vaccine has been similarly developed (Gao et al., 2020a), expanded in Vero cells and chemically inactivated by using β-propiolactone and mixed with alum adjuvant (Gao et al., 2020a) (Figure 3A). In clinical trials, safety, tolerability and immunogenicity have been also reported for the CoronaVac vaccine (Zhang et al., 2021b; Han et al., 2021; Tanriover et al., 2021; Wu et al., 2021). Bharat Biotech together with the Indian Council of Medical Research and the National Institute of Virology have developed the BBV152 vaccine (Covaxin) (Sharma et al., 2021; Yadav et al., 2021). This inert, safe and quite efficient virus vaccine has been obtained from the NIV-2020-770 isolate, which presents the emerged D614G mutation that increases infectiveness and case fatality rate (Becerra-Flores and Cardozo, 2020; Zhang et al., 2020c; Eaaswarkhanth et al., 2020; Korber et al., 2020; Lorenzo-Redondo et al., 2020; Daniloski et al., 2021; Mansbach et al., 2021; Müller et al., 2021; Plante et al., 2021), and it was formulated with imidazoquinoline (IMDG; a toll-like receptor 7/8 agonist molecule (Miller et al., 2020)) and absorbed in Alum-gel (Algel) as adjuvants (Ella et al., 2021a; Ella et al., 2021b; Sharma et al., 2021; Yadav et al., 2021).

Another nano-based vaccination strategy consist in exposing the immunogenic viral antigen at the surface of empty spherical non-infectious structures named VLPs (viral like particles) (Al-Barwani et al., 2014a; Donaldson et al., 2018), which could be named or classified in enveloped and non-enveloped VLPs (eVLPs and neVLPs, respectively) [reviewed in (Nooraei et al., 2021)] (Figure 3A). VLPs are obtained *in cellulo* by the self-assembly mechanism of viral proteins, during the expression of the immunogenic protein of interest (Zeltins, 2013; Al-Barwani et al., 2014a). VLP-based vaccines could be considered a kind of subunit vaccines, presenting therefore a characteristic immune response associated with exogenous antigens (Garcea and Gissmann, 2004; Moser et al., 2011; Salvador et al., 2011; Lua et al., 2014; Nooraei et al., 2021). VLPs could be chemically modified by conjugating receptor ligands onto the surface of VLPs to drive their binding to and uptake into APCs expressing the corresponding receptor (Al-Barwani et al., 2014b; Nooraei et al., 2021). Polysorbate 80, sodium borate/phosphate, L-histidine, and 2-phenoxyethanol are often used in the formulation of VLP-based vaccines as a surfactant stabilizer, preservative and buffering agents (Lua et al., 2014) (Figure 3A). VLP-derived vaccines are significantly safer than live-attenuated or inactivated virus vaccines, as well as subunit or particulate vaccines, due to the noninfectious nature of the VLPs (Plummer and Manchester, 2011; Lua et al., 2014; Alon et al., 2017). In fact, some commercially successful vaccines to immunize against human papillomavirus (HPV), hepatitis B virus (HBV) and hepatitis E virus (HEV) are VLP vaccines (Block et al., 2006; Monie et al., 2008; Donaldson et al., 2018; Lee et al., 2019; Qian et al., 2020a). Therefore, VLPs are resembling natural virions but being empty and bearing at the surface membrane of the assembled particle the immunogenic protein (Zeltins, 2013; Al-Barwani et al., 2014a; Donaldson et al., 2018; Mohsen et al., 2020). In the case of COVID-19 vaccine developments, it bears the entire S glycoprotein of SARS-CoV-2 or just the recombinant RBD region of the S protein (Geng et al., 2021; Prates-Syed et al., 2021; Yilmaz et al., 2022). Promising results have been obtained with a VLP-based RBD vaccine which provide highly effective protection against SARS-CoV-2 infection in a mouse model (Geng et al., 2021). Likewise, a VLP-based vaccine carrying an hexaproline prefusion-stabilized S viral protein (S-6p) (Hsieh et al., 2020) has been developed with excellent results in several animal models for SARS-CoV-2 infection (i.e., mice, rats and ferrets) (Yilmaz et al., 2022). This VLP vaccine contains alum and a K-type synthetic oligodeoxynucleotides (ODN) containing unmethylated CpG (5′-Cytosine-phosphate-Guanine-3′) motifs (CpG ODN) as a vaccine adjuvant (Klinman et al., 2009; Ezoe et al., 2020) to promote cellular and humoral immunity (Yilmaz et al., 2022) (Figure 3A). This CpG ODN/alum-adjuvanted 6p-VLP vaccine (VLP-58-1023-Al-K3) is currently being evaluated in a clinical trial (NCT04818281). Furthermore, some VLP developments include other SARS-CoV-2 proteins to stabilize the VLP, such as M (membrane), E (envelope) and N (nucleocapsid) proteins, thereby favoring assembling and expression of the immunogenic S protein (Xu et al., 2020b; Plescia et al., 2021). Worth of mention is the Novavax subunit vaccine (NVX-CoV2373) that uses a recombinant modified nanoparticle S protein of SARS-CoV-2 (Shin et al., 2020b; Chauhan et al., 2020; Keech et al., 2020; Formica et al., 2021; Heath et al., 2021; Tian et al., 2021; Dunkle et al., 2022) together with a Matrix-M adjuvant (Bengtsson et al., 2013) assembled in non-enveloped VLPs (neVLPs) (Bangaru et al., 2020) (Figure 3A). As indicated above, the immunogenic S protein used in this NVX-CoV2373 vaccine is a S-2P protein (1-1273 amino acids) stabilized in the prefusion state, expressed using a baculovirus/insect cell system and then incorporated into polysorbate 80 detergent (Bangaru et al., 2020) (Figure 3B). This full-length S protein has been modified in the polybasic domain at the R682RAR685 sequence, mutated by the Q682QAQ685 sequence (Bangaru et al., 2020; Keech et al., 2020; Tian et al., 2021) (Figure 3B), to avoid its processing by the host furin proteinase (Hoffmann et al., 2020a; Andersen et al., 2020; Shang et al., 2020b; Walls et al., 2020b; Jaimes et al., 2020; Johnson et al., 2020; Lemmin et al., 2020; Johnson et al., 2021; Whittaker, 2021; Villoutreix et al., 2022). The NVX-CoV2373 is a safe vaccine against SARS-CoV-2 infection (Heath et al., 2021), commercially successful as a COVID-19 vaccine (Mahase, 2021; Shinde et al., 2021). The Radbound University and AdaptVac (a company part of the EU H2020 funded PREVENT-nCoV consortium) have also developed a promising neVLP SARS-CoV-2 vaccine (ABNCoV2 vaccine) based on the S RBD (Fougeroux et al., 2021) (Figure 3A), which is currently in phase 1/2 clinical trials (NCT04839146) and phase 3 results are expected by the end of 2022.

On the other hand, recombinant protein-based vaccines are promising candidates for immunization because they offer a safety profile with no live or attenuated viral components in its formulation, avoid potential genomic integration issues, since there are no genomes to be delivered into host cells, and seem to be suitable for immunocompromised individuals (Pollet et al., 2021a; Kleanthous et al., 2021; Kyriakidis et al., 2021) (Figure 3A). The above presented Novavax NVX-CoV2373 vaccine could be also considered as a recombinant protein-based vaccine (Bangaru et al., 2020; Heath et al., 2021; Mahase, 2021; Shinde et al., 2021), as well as some developments focused on the RBD sequence of the S1 subunit of the viral S protein (Malladi et al., 2021b; Kleanthous et al., 2021; Lee et al., 2021) (Figure 3A). The RBD fragment has been assayed as a protein-recombinant vaccine candidate by delivering soluble monomers or dimers of the RBD region (Yang et al., 2020a; Dai et al., 2020; Dalvie et al., 2021a; Malladi et al., 2021a; Malladi et al., 2021b; Pollet et al., 2021b; Chen et al., 2021c; Wang et al., 2021f; Sun et al., 2021), fused to NPs (Walls et al., 2020a; Ma et al., 2020; Arunachalam et al., 2021; Kim et al., 2021b; He et al., 2021; King et al., 2021; Eugenia Toledo-Romaní et al., 2022) or exposed on VLPs (Dalvie et al., 2021a; Dalvie et al., 2021b; Kang et al., 2021; Tan et al., 2021; Zha et al., 2021) (Figure 3A). These recombinant protein-based vaccines present several advantages for development and distribution, since they are produced in large scale, their designs and formulations are safe and immunogenic as those developed using the full-length S protein, they are temperature-stable vaccines and doses have an affordable cost (Malladi et al., 2021b; Kleanthous et al., 2021; Lee et al., 2021). Remarkably, one the most promising recombinant protein vaccines is the new PHH-1V vaccine by HIPRA for intramuscular administration, developed in a consortium with different research centers and Hospitals (Barreiro et al., 2022; Corominas et al., 2022; Leal et al., 2022) (Figure 3A). The PHH-1V vaccine is a highly purified RBD heterodimer (i.e., to avoid limited immunogenicity obtained with monomeric RBD protein (Dai et al., 2020; Yang et al., 2021b)) which is formulated with SQBA, an oil-in-water adjuvant produced by HIPRA, and prepared as an emulsion that does not need to be reconstituted or thawed before administration (Figure 3A). Importantly, the PHH-1V vaccine consists in a fusion heterodimer that contain S-RBD sequences from two SARS-CoV-2 VOCs (Barreiro et al., 2022; Corominas et al., 2022; Leal et al., 2022), B.1.1.7 (Alpha) and B.1.351 (Beta). PHH-1V RBD sequences carry key mutations of epidemic relevant VOCs of SARS-CoV-2, such as the K417N, E484K and N501Y22 humoral escape mutations (Wang et al., 2022a; Wang et al., 2022c), in order to elicit humoral and cellular immune responses against these VOCs or future emerging variants bearing these escape mutations. Hence, the RBD of the S glycoprotein of B.1.351 (Beta), P.1 (Gamma) and B.1.621 (Mu) variants present E484K and N501Y mutations (Wang et al., 2022a; Wang et al., 2022c), whereas both K417N and N501Y immune escape mutations (i.e., for natural and vaccine elicited immunity) are characteristics of the B.1.351 (Beta) and the currently predominant Omicron (B.1.1.529 and BA lineages) variants (Chen et al., 2021a; Wang et al., 2022a). The European Commission has signed a joint procurement contract with the Spanish HIPRA pharmaceutical firm for the supply of the PHH-1V recombinant protein COVID-19 vaccine, which will be commercially available once the European Medicines Agency (EMA) approve it.

COVID-19 vaccines are not protective against SARS-CoV-2 infection and reinfections, due to the absence of antigen specific immunity elicited in the nasal cavities (considered the main route of entry of SARS-CoV-2 into the organism together with oral cavities and ocular conjunctiva) (Cantuti-Castelvetri et al., 2020; Daly et al., 2020; Gengler et al., 2020; Bougakov et al., 2021; Gallo et al., 2021; Huang et al., 2021; Meinhardt et al., 2021; Rodríguez-Ares et al., 2021; Maurin et al., 2022; Pipolo et al., 2022) and the associated mucosal epithelial cells, such as ciliated cells and mucus-producing goblet cells (Lee et al., 2020a; Hou et al., 2020; Osan et al., 2020; Wölfel et al., 2020; Ahn et al., 2021; Bougakov et al., 2021; Meinhardt et al., 2021; Nakayama et al., 2021; Robinot et al., 2021; Morrison et al., 2022; Osan et al., 2022; Pipolo et al., 2022). In these tissues, viral infection is cleared by nasal-associated lymphoid tissue (NALT), including lymphocytes (B and T cells), dendritic cells (DCs), and macrophages. However, these immune responses are not protecting against reinfections (reviewed in (Gallo et al., 2021)). Therefore, sterilizing vaccines should promote mucosal immunity at that level, in order to render nasal cavities and NALT as barriers for SARS-CoV-2 infection and reinfections (reviewed in (Focosi et al., 2022)). In this regard, promising results have been obtained in animal models with the NSP16-deficient SARS-CoV-2 virus, which acts as a live attenuated vaccine that confers sterilizing immunity after intranasal administration of a single dose (Ye et al., 2022). Other studies also reported positive results in animal models (mice, golden hamsters, and ferrets) for nasal-mucosal, inhaled vaccination conferring long-lasting systemic and mucosal immunity (Moreno-Fierros et al., 2020; Mudgal et al., 2020; Ashraf et al., 2021; Park and Lee, 2021; Tiboni et al., 2021; Focosi et al., 2022; Heida et al., 2022). These vaccine developments would achieve the desired sterilizing immunity for mucosal cells and tissues against SARS-CoV-2, just at the door of our organism for viral infection. However, intranasal or mucosal vaccines should follow the tissular dynamics followed by SARS-CoV-2 to accomplish neutralizing immunity, and therefore all designed vaccines and associated adjuvants must be safe, avoiding reaching and affecting the central nervous system (CNS) via the olfactory bulb that could compromise the life of the inoculated individuals. Fortunately, for vaccine developments with SARS-CoV-1 S protein, it has been reported that the use of rAd vectors via sublingual administration induce systemic and mucosal immunity without redirection of the viral rAd vector to the brain (Shim et al., 2012). Similar immunoprotection results have been obtained with a recombinant adeno-associated virus (rAAV) expressing the RBD of the SARS-CoV-1 S protein (RBD-rAAV) intranasal administered (Du et al., 2008). Although these studies show that mucosal immunization against SARS-CoV-1 S protein did not provide full sterilizing immunity, they prevent virus dissemination to the lung and prevent respiratory distress, being appropriated models to achieve SARS-CoV-2 sterilizing vaccines. Moreover, the use of appropriate nanoreagents to form stable and safe nanoparticles for intranasal administration of Abs, proteins or genomic fragments as immunogens would be key to accomplish the desired sterilizing immunity. Thus, a recent study has reported that it is possible to deliver a mRNA to the lung via nebulization of LNPs made of lipids, neutral or cationic helper lipids and polyethylene glycol (PEG). The intranasal administration of nebulized LNPs carrying a mRNA encoding for a broadly nAb targeting haemagglutinin protects mice from lethal challenge of the H1N1 subtype of influenza A virus (Lokugamage et al., 2021).

Therefore, nano-based COVID-19 vaccine designs use different nanomaterials, nanocarriers and technologies to enhance the bioavailability of the viral immunogen, control its delivery and faster uptake by target host cells and tissues, and also acting as adjuvants to activate the immune responses against the viral antigen, in order to promote specific and protective immune activities against SARS-CoV-2 infection and severe COVID-19 disease (reviewed in (Campos et al., 2020; Chauhan et al., 2020; Chung et al., 2020; Talebian and Conde, 2020; Constantin et al., 2021; Creech et al., 2021; Machhi et al., 2021; Rashidzadeh et al., 2021; Varahachalam et al., 2021; Guerrini et al., 2022; Souri et al., 2022)) (Figure 3).

### 2.3 Virosurveillance: Genomics to trace SARS-CoV-2

Surveillance of infectious diseases, and particularly of emerging and new emerging virus diseases, is key for ensuring community and global health in the XXI century. In 7 years from 2011, WHO alerted and followed nearly 1,500 epidemic events in several countries due to emerging viruses, such as SARS, MERS, influenza, Zika virus (ZIKV), Yellow Fever virus (YFV), and Ebola virus (EBOV), among other viruses (Global-Preparedness-Monitoring-Board-(GPMB), 2019). The world began to realize the fragility of our societies and economies in facing severe non-curable and/or lethal emerging virus when the 2014–2015 ZIKV pandemic seriously affected two of the biggest world sports events: the Olympic Games in Brazil (2016) and the World Soccer Championship in Brazil (2018) (Petersen et al., 2016; Salvador and Fujita, 2016). These epidemic events announce the new global era the world is facing with fast-spreading outbreaks, difficult to manage and having impact on lives, health systems, administration and the economy of companies, families and countries, as is occurring nowadays with the worldwide SARS-CoV-2 infection (Reports, 2020) responsible for the still ongoing COVID-19 pandemic (Beech and Anseel, 2020; Brammer and Clark, 2020; Nicola et al., 2020; Shankar, 2020; Verbeke, 2020; Heinberg and Steffen, 2021; Klebe et al., 2021; Onyeaka et al., 2021; Poudel et al., 2021; Shang et al., 2021; Sheng et al., 2021; Sohrabi et al., 2021; Yen et al., 2021; Chang et al., 2022; Desai et al., 2022). Surveillance of these emerging viruses involves producing information to guide actions at different levels (Buckeridge and Cadieux, 2006) to protect all sectors that are in danger because of the absence of preparedness policies (Global-Preparedness-Monitoring-Board-(GPMB), 2019; Lee et al., 2020c). Therefore, surveillance is a fundamental tool for public health and our societies, where genomics for virus-surveillance has gain relevance as the worldwide tracking system of the different emerged SARS-CoV-2 variants, as it has been demonstrated in different countries. A remarkable example is the first WGS obtained from patients’ viral samples. A few days passed from the first observed Wuhan patients, affected by the new unknown disease, to the world-wide communication of the viral sequence (Ackermann et al., 2020; Cao et al., 2020; Lu et al., 2020).

**FIGURE 4:**
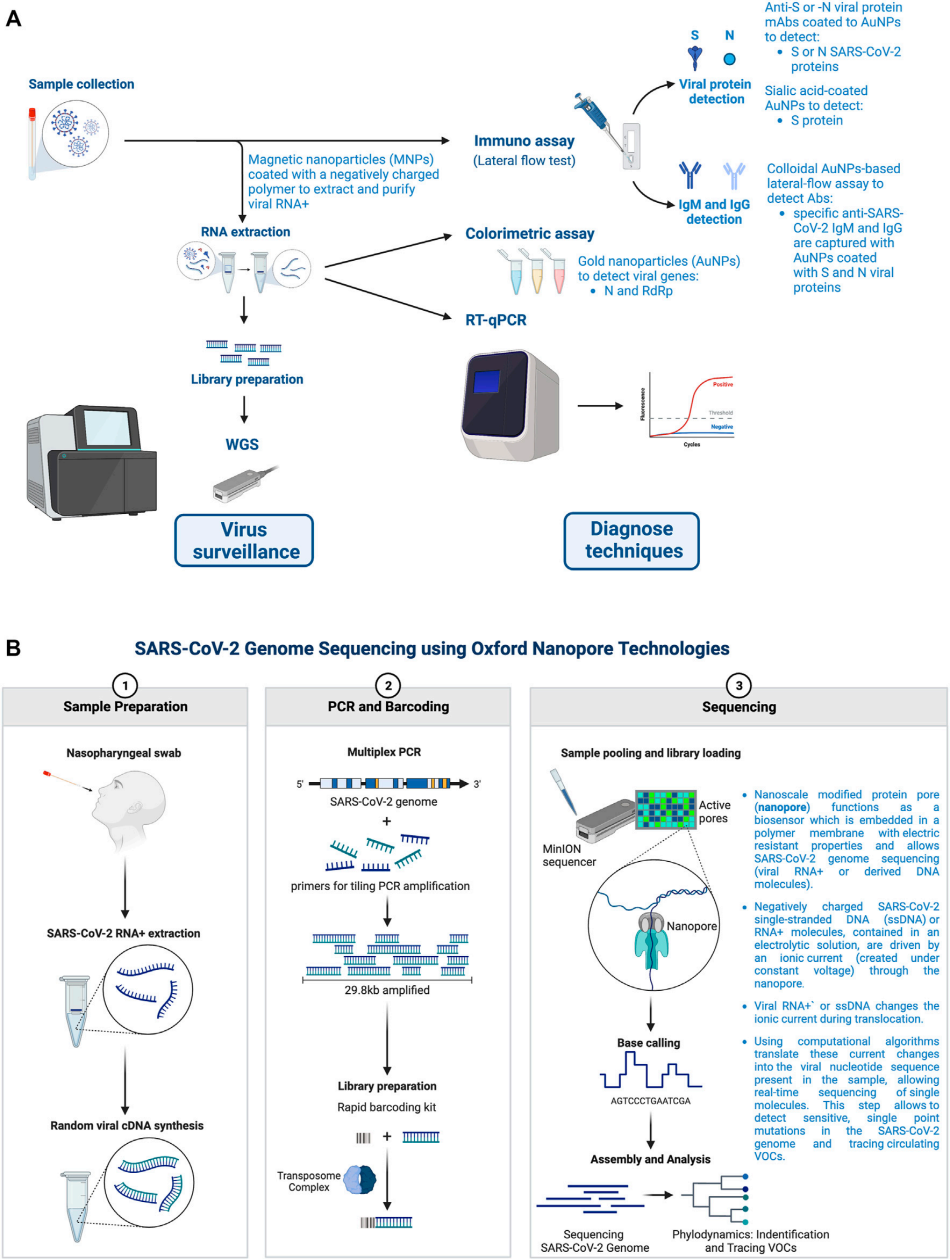
Nanotechnology and reagents used for genome sequencing technologies and serological approaches for SARS-CoV-2 surveillance and diagnose. **(A)** Viral RNA+ extraction from patients’ samples have allowed to prepare a library or primers for WGS that is very useful for virus surveillance and the detection of VOCs bearing key mutations associated with immune responses escape and vaccine breakthrough. Likewise, viral RNA+ extraction have permitted to develop specific, sensitive, scalable, rapid and low-cost RT-qPCR tests for diagnose, both in hospitals and in PoC facilities. Nanomaterials used for RNA+ isolation and purification that allow scalation of RT-qPCR and other gene-based diagnose tests are indicated. Moreover, nanotechnology and nanoreagents used for serological lateral flow tests permit PoC detection of the viral S and N antigens (i.e., from saliva or anterior nasal swabs (nasopharyngeal exudate or other upper/lower respiratory samples)) or Igs (IgM, IgG and IgA) against SARS-CoV-2 S and N proteins from patient blood samples. All nanomaterials and associated strategies (i.e., lateral flow test, colorimetric test, and RT-qPCR) to detect viral genomes and proteins are shown. **(B)** SARS-CoV-2 genome sequencing using ONT (Oxford nanopore technology). Rapid sequencing of the SARS-CoV-2 is key for a correct public health response during a pandemic. Through nanopore sequencing, RNA samples are retrotranscribed (1), and the whole genome amplified with various sets of primers (2). Amplified fragments can incorporate barcodes through a transposase (2) and then samples can be pooled and sequenced through nanopores (3). SARS-CoV-2 RNA+ is also read directly by nanopore. Detected current patterns over time define the specific nucleobases in a sequence (3), which can be demultiplexed, assembled and analyzed, thus allowing the detection of sensitive, single point mutations in the viral genome and tracing circulating VOCs (3). Designs and templates are created with BioRender.

The knowledge of a complete new CoV genomic sequence (available at GISAID and GenBank (accession number MN908947) (Cao et al., 2020; Lu et al., 2020)), designated as Wuhan-Human 1 CoV (Wuhan-Hu-1 or WHCV) (Ackermann et al., 2020), allowed the rapid development of probes, reagents and tools for specific and sensitive detection of viral sequences in the patients infected by SARS-CoV-2 (Cheng et al., 2020a; Corman et al., 2020; Dutta et al., 2022). One of the first epidemiological parameters that these tools helped to assess was the basic reproduction number or R_0_ (R naught) value for virus community transmission, estimated from 2.2 to 2.68 for the ancestral Wuhan-Hu-1 strain (Liu et al., 2020e). These tools allow: 1) specific diagnosis and virosurveillance of outbreaks and community virus spreading, 2) to follow how the virus is evolving and the potential relationships between the genome viral sequence, 3) its mutations, virus infectiveness and disease severity, and 4) to understand natural and vaccine-elicited immunity breakthrough events that help to adapt new vaccines and drugs (Alcoba-Florez et al., 2020a; Corman et al., 2020; Ciuffreda et al., 2021a; Alcoba-Florez et al., 2021; Ciuffreda et al., 2022). A historical and paradigmatic genomic virosurveillance global action, near to real-time and an unprecedented scale (Gardy et al., 2015; Lucas et al., 2020a), started after at that moment by fostering a continuous communication and classification of the obtained viral sequences through different resources (e.g., GISAID, GenBank, Nextstrain, Pangolin, among others) (Hadfield et al., 2018; Andersen et al., 2020; Liu et al., 2020d; Khare et al., 2021; O'Toole et al., 2021; Sayers et al., 2021; Underwood et al., 2022)), where WGS, GWAS (genome-wide association study) and nanopore sequencing approaches and technologies have gain relevance for surveillance and diagnose (Arora et al., 2022) (Figure 4).

WGS provides a deep insight into the DNA sequence of the viral genome and, when combined with metadata analysis at the individual or community level, it has allowed to analyze the origins of the COVID-19 pandemic (Al Kaabi et al., 2021; Singh et al., 2021; Costa et al., 2022), and to characterize outbreaks in a community or region (Alcoba-Florez et al., 2021; Aggarwal et al., 2022; Ciuffreda et al., 2022), hospitals (Rivett et al., 2020), farms (Badiola et al., 2021; Oude Munnink et al., 2021), schools (Huang et al., 2022) and homes (Ladhani et al., 2022), among other social or industrial entities (Bartik et al., 2020; Murti et al., 2021) (Figure 4A). Moreover, public health responses to the COVID-19 pandemic are supported by sequencing WGS from circulating SARS-CoV-2 VOCs and analyzing the risk host factors. WGS enables mapping of community and international viral transmission, thereby identifying and tracking the SARS-CoV-2 emerging variants (Gohl et al., 2020; Rivett et al., 2020; Aggarwal et al., 2022; Obermeyer et al., 2022; Rosenthal et al., 2022) (Figure 4A), even helping in clinical care using samples collected from patients-rapid antigen test devices (Gohl et al., 2020; Arora et al., 2022; Rosenthal et al., 2022). Host genetics is also a key element in this scenario. WGS and genome-wide association studies (GWAS) have been used to identify host genetic factors that predict COVID-19 severity and patient survival. In this regard, it was of special interest the GWAS analysis associating inborn errors of immunity (i.e., mainly INF and TLR genes and associated pathways) with severe COVID-19 (Bastard et al., 2020; Chung et al., 2020; Ellinghaus et al., 2020; Grasselli et al., 2020; The-Covid-Host-Genetics-Initiative, 2020; Al Kaabi et al., 2021; Alcoba-Florez et al., 2021; Bastard et al., 2021; Castano-Jaramillo et al., 2021; Fallerini et al., 2021; Niemi et al., 2021; Pairo-Castineira et al., 2021; Tan et al., 2021; Karlsen, 2022; Loucera et al., 2022; Oh and Shin, 2022). In these critically ill patients, the SARS-CoV-2 infection have generally been diagnosed mainly by specific and sensitive retrotranscription and quantitative polymerase chain reaction (RT-qPCR) technique (COVID-19-Host-Genetics-Initiative, 2021) (Figure 4A). In fact, this quantitative, real-time PCR allows to identify specific features in some concerning variants, like S-gene target failure (SGTF) found in the B.1.1.7 (Alpha) and Omicron variants, further confirmed by S-gene sequencing (Volz et al., 2021; Aggarwal et al., 2022; Wolter et al., 2022). In other studies, single-cell RNA sequencing data has been combined with viral WGS analysis, leading key results corelating the level of expression of CCR1 and CXCR6 chemokine receptors on CD8^+^ T cells and monocytes with COVID-19 severity (Alsved et al., 2022). The presence of SARS-CoV-2 VOCs are detected by genomic sequence analysis. It was used to identify the first VOCs B.1.1.7 (Alpha), B.1.351 (Beta) and P.1 (Gamma) and those that emerged later on, such as B.1.617.2 (Delta) and Omicron BA.1 and BA.2 lineages associated with enhanced viral transmission and pathogenicity, and immune escape (Akilesh et al., 2021; Alcoba-Florez et al., 2021; Altmann et al., 2021; Hirotsu and Omata, 2021; Nakamichi et al., 2021; Alsafar et al., 2022; Ciuffreda et al., 2022; Harrigan et al., 2022; Lyngse et al., 2022; Wawina-Bokalanga et al., 2022). In fact, the viral WGS is appropriated for the identification of the viral genomic variants that are associated with severe disease patterns (Hoffmann et al., 2021; Hoang et al., 2022; Overton et al., 2022; von Wintersdorff et al., 2022).

One sequencing approach that has gained relevance in the context of real-time surveillance of SARS-CoV-2 entails the long-read sequencing technology from Oxford Nanopore Technologies (ONT) (Corman et al., 2020; Pater et al., 2021). ONT technology relies on a nanoscale modified protein pore, named nanopore, that functions as a biosensor which is embedded in a polymer membrane with electric resistant properties (Deamer et al., 2016; van Dijk et al., 2018) (Figure 4B). Negatively charged single-stranded DNA (ssDNA) or RNA molecules, contained in an electrolytic solution, are driven by an ionic current (created under constant voltage) through the nanopore. These charged nucleic acid molecules move from the negatively charged “cis” side to the positively charged “trans” side of the nanopore (Figure 4B). Importantly, a motor protein that acts as an anchored bar pass the ssDNA or RNA molecules through the nanopore in a controlled manner thereby reducing the translocation speed. RNA or ssDNA changes the ionic current during translocation. Using computational algorithms translate these current changes into the nucleotide sequence present in the sample, allowing real-time sequencing of single molecules (Figure 4B). Moreover, the motor protein presents a helicase activity that unwound the double-stranded DNA (sdDNA) or RNA-DNA duplexes into single-stranded molecules that pass through the nanopore (reviewed in (Deamer et al., 2016; Wang et al., 2021e)). Previously, for assuring SARS-CoV-2 genome sequencing and considering that viral RNA extracted from patients samples could be degraded and not the best quality, a series of shorter amplicons (i.e., 400 nucleotides) are used for amplifying across the virus genome and cover the whole sequence (Figure 4B), thereby the viral RNA is converted into DNA by reverse transcription and PCR is used for amplicons generation to cover all the sequence and identifying overlapping amplicons (Kim et al., 2020a; Bull et al., 2020; Brejová et al., 2021; Stüder et al., 2021; Wang et al., 2022b; Li et al., 2022; Xu et al., 2022) (Figure 4B). Then, samples were barcoded by using specific kits and following ligation with a sequencing adapter, in order to generate a library for nanopore sequencing (Figure 4B). The protocol for nanopore sequencing of tiled PCR-generated amplicon pools has been developed an adapted by the Artic Network (https://artic.network/). These procedures have been used for other emerging RNA viruses and microorganisms (Quick et al., 2016; Quick et al., 2017; Ciuffreda et al., 2021b). ONT or nanopore sequencing offers several advantages to obtain viral WGS because these devices do not require large supporting laboratory infrastructures, are portable and are inexpensive, allowing rapid sequencing analysis to be scalable and at reach for any laboratory (Coloma et al., 2016; Corman et al., 2020; Ciuffreda et al., 2021b). Nanopore sequencing allows to detect single nucleotide variants and small insertion/deletions in circulating SARS-CoV-2 with high accuracy and high sensitivity (Corman et al., 2020; Noh et al., 2020; Baker and Dolgin, 2021; Ciuffreda et al., 2021b; González-Recio et al., 2021; Pater et al., 2021), as for the key mutations in SARS-CoV-2 VOCs (La Rosa et al., 2021) (Figure 4B). Moreover, nanopore technology allows to directly sequence the SARS-CoV-2-RNA + genome from patients’ samples (Viehweger et al., 2019; Kim et al., 2020a; Vacca et al., 2022; Xu et al., 2022) (Figure 4B). Nanopore sequencing combined with a machine learning algorithm permits rapid hospital diagnoses of SARS-CoV-2 from saliva samples of the patients (Horejs, 2021). Nanopore sequencing could be used in SARS-CoV-2 diagnose with other technologies, such as the reverse transcription (RT) loop mediated isothermal amplification (LAMP; (Notomi et al., 2000)) (RT-LAMP; (Huang et al., 2022)) underlying the LamPORE approach (James et al., 2020). LamPORE is considered a high-throughput substitute of RT-qPCRs for rapid and accurate diagnosis of SARS-CoV-2 infection (Peto et al., 2021; Ptasinska et al., 2021) while used for SARS-CoV-2 genotyping (Morsli et al., 2021). Moreover, ONT sequencing has been applied for accurate tracking of intra-hospital SARS-CoV-2 transmission (Løvestad et al., 2021). Thus, this affordable and easy-to-scale viral sequencing technology has been a widespread solution for tracking SARS-CoV-2 VOCs (Rios et al., 2021; Yakovleva et al., 2021; Barbé et al., 2022), and help decision making for local public health agents without the need of large genomic infrastructures.

The ability of SARS-CoV-2 to evolve forces constant virosurveillance and the adaptation of the required tools, especially at the genetic level for tracking emergence of new VOCs (Lucas et al., 2020a). The rapid detection and characterization of new variants is therefore crucial to informing the potential efficacy of vaccines and therapeutics (Lucas et al., 2020a). The next-generation genome sequencing technologies, as well as nanopore allow to rapidly adapt protocols and reagents for accurate and sensitive detection of new emerging SARS-CoV-2 VOCs (Figure 4).

### 2.4 Point-of-care: Rapid diagnosis and control of outbreaks in the field

Point-of-care (PoC) facilities and detection technologies have been stablished all around the world because they enable decentralized (i.e., from hospitals), rapid, sensitive, low-cost diagnosis of SARS-CoV-2 infection in neighborhoods health offices and identification of COVID-19 symptoms (Kilic et al., 2020; van Dongen et al., 2020; Valera et al., 2021). The PoCs have accomplished a crucial role in epidemiology surveillance and *in situ* first triage of ill patients, avoiding sample transport to a clinical laboratory for analysis, thereby easing Hospital work and management of more sever COVID-19 patients (Vandenberg et al., 2021). PoCs have been of importance for prevention of SARS-CoV-2 transmission to the community and to determine who requires quarantine. In fact, the above reported, first viral genetic sequences enabled the rapid development of PoC real-time RT-qPCR diagnostic tests specific for the new CoV (Kampf et al., 2020a; Corman et al., 2020; Hoehl et al., 2020; Rothe et al., 2020; Gupta et al., 2021a) (Figure 4A). In these PoCs the technology used for testing patients permits minimum samples processing, the use of saliva or anterior nasal swabs (nasopharyngeal exudate or other upper/lower respiratory samples) for accurate SARS-CoV-2 detection (i.e., viral RNA or mainly S and/or N antigen proteins), as well as Abs able to recognize viral proteins, either in symptomatic or asymptomatic patients (Figure 4B). Therefore, COVID-19 tests are developed for molecular diagnostics (viral genome), antigen (viral proteins) and antibody tests (Kilic et al., 2020; Valera et al., 2021). For personnel using these tests, it is important to know the accurate protocol for taking samples (i.e., it is key to have skills to know the most appropriate moment for taking samples) and use of these techniques, in order to minimize non-specific results (false positives or negatives reads) due to inappropriate handling or inadequate interpretation of the obtained results, since this information is considered together with the symptomatology of patients for making clinical decisions (Jacofsky et al., 2020).

Infected individuals have been determined by using gold-standard COVID-19 diagnosis to detect SARS-CoV-2 genomic fragments in patients’ samples, associated with viral proteins, such as the S glycoprotein, N, E and the RNA-dependent RNA polymerase (RdRp), and using RNA purification and one-step RT-qPCR (Alcoba-Florez et al., 2020a; Alcoba-Florez et al., 2020b; Khailany et al., 2020; Alcoba-Florez et al., 2021) (Figure 4A, *see RT-qPCR schemes*). As presented above, the Chinese authorities published in GenBank and in the GISAID portal database the complete genome of SARS-CoV-2. Thanks to this contribution, the microbiological diagnosis of COVID-19 has been based on the detection of the viral genetic material (RNA+) of SARS-CoV-2 from the collection of respiratory samples from patients with compatible symptoms (Corman et al., 2020; Dutta et al., 2022). Various laboratories began development for the detection of SARS-CoV-2 in accordance with WHO guidelines, which initially published five protocols for diagnosis using RT-qPCR (Corman et al., 2020; Dutta et al., 2022). The first validated RT-qPCR detection test was developed by the Berlin Institute of Virology (Corman et al., 2020). The laboratory PCR procedure consists in the extraction of nucleic acid and the amplification reaction. These processes could be automated for rapid diagnose and to reduce errors (Cheng et al., 2020a) (Figure 4A, *see RT-qPCR schemes*). Moreover, this RT-qPCR technique could be adapted to rapidly detect SARS-CoV-2 (i.e. by 1 h), bypassing the time consuming RNA extraction step and accelerating viral detection, without test sensitivity reduction and allowing sample scaling to increase testing capacity by sample pooling, being of importance during severe outbreaks with large number of people to be tested in a short period of time (Alcoba-Florez et al., 2020a; Alcoba-Florez et al., 2020b; Alcoba-Florez et al., 2021). Likewise, functionalized coated magnetic nanoparticles (MNP) with a negatively charged polymer (i.e., created from 3-aminopropyl triethoxysilane (APTES) polymerized with diacrylate-amine to obtain the poly (amino-ester), poly-NH2-MNPs) allows viral RNA extraction and purification, in order to implement the detection of the SARS-CoV-2 virus by RT-qPCR on a large scale (Chacón-Torres et al., 2020) (Figure 4A).

Serological tests are diagnostic methods used for rapid detection of Immunoglobulins (Igs) (i.e., IgG, IgM and IgA) against SARS-CoV-2 S and N proteins (Dinnes et al., 2020; Tuaillon et al., 2020; Yamayoshi et al., 2020). These tests are named “rapid tests” (about 15 min) and consist in immunochromatographic lateral flow assays to detect Abs that indicate that individuals are infected or have been recently infected by SARS-CoV-2 (Figure 4, *see lateral flow test schemes*). Serological tests for detection of anti-SARS-CoV-2 Abs to support infected individuals diagnose were first used by the Duke-NUS Medical School in Singapore. This test, named cPass™, was developed in a consortium with GenScript Biotech Corporation and A*STAR, and measures nAbs in an hour (Jung et al., 2021). Lateral flow Ab tests for SARS-CoV-2 require a drop of blood from the patient, usually from a finger prick, similar to tests to monitor blood sugar in certain types of diabetes. This capture and binding process produces a color change along the test and control lines that can be seen with the naked eye, producing one, two, or three lines depending on the type of antibodies (Abs) present (IgM or IgG) (Figure 4A, *see lateral flow schemes*) (Figure 4A). These tests work very differently from the RT-qPCR technique and focus on detecting the patient’s immune response to the virus rather than detecting the virus itself. These Ab tests require knowledge about the structure and key infection epitopes of the viral proteins, specifically, those proteins to which the immune system responds by triggering the production of nAbs that could neutralize the virus (Guo et al., 2020b; Jiang and Zhang, 2020; Zhao et al., 2020). In this matter, the generation of Abs against SARS-CoV-2 begin about the sixth day of the onset of symptoms, when a decrease in viral load could be observed. Half of the infected cases have total Abs around the seventh day post-infection and 15 days after infection, almost 100% of infected individuals presented Abs both in mild and severe cases (Guo et al., 2020b; Jacofsky et al., 2020; Long et al., 2020; Zhao et al., 2020). These Ab-based techniques seek to detect the immune response of patients, which increases as the infection progresses, and therefore offer the possibility to detect active disease (Jacofsky et al., 2020). Of note, the presence of Abs does not exclude the possibility of continuing to be contagious and is not indicative of having sterilized immunity against recurrent SARS-CoV-2 infections, either in vaccinated or in vaccinated people where reinfections frequently occur (Bergwerk et al., 2021; Gupta et al., 2021b; Hagan et al., 2021; Song et al., 2021; To et al., 2021; Acharya et al., 2022; Kuhlmann et al., 2022; Ren et al., 2022; Singanayagam et al., 2022).

Antigen serological tests are based on the detection of viral proteins specific to SARS-CoV-2, such as the N protein and the S1 or S2 subunits of the S viral glycoprotein (reviewed in (Falzone et al., 2021; Li and Li, 2021)) (Figure 4A, *see lateral flow tests for immune assays*). Considering that viral replication is more accentuated in the acute phase, the antigenic test should be carried out in the first five-seven days after the onset of symptoms. The biological samples used to detect viral proteins come from nasopharyngeal, oropharyngeal or sputum exudates (reviewed in (Falzone et al., 2021; Li and Li, 2021)). The test contains a strip of nitrocellulose paper where a line has been painted with Abs that adhere to the virus (Figure 4, *see lateral flow test schemes*). The sample is mixed with a diluent to be dispensed into the test in the window intended for the sample. In this sample-window, the sample is met by colloidal gold- or latex-labeled Abs that make them visible. Later, in the test line (T), more Abs (unmarked) are placed that will serve to confirm whether or not there is an infection. It seems that the viral load is higher in sputum and nasopharynx, being higher in the initial stages of infection, but if the sample viral load is in the limit of detection, false negative reads will occur, instead the person is infected (reviewed in (Falzone et al., 2021)).

Innovative tools for SARS-CoV-2 detection have also been developed using nanomaterials and derived technology. Multiple characteristics have made these nanomaterials good candidates for diagnosis support: the variability in size, structure and geometry, the surface reactivity that have allowed their functionalization with other biomolecules, the electrical conductivity and/or magnetic properties or the quantum or fluorescent characteristics (Kang et al., 2009; Ansari et al., 2010; Cao et al., 2011; Holzinger et al., 2014; Sun et al., 2017; Mitchell et al., 2021). For example, gold nanoparticles (AuNPs), magnetic NPs and quantum dots (QDs) have been engineered to detect SARS-CoV-2 genes and proteins, as well as anti-SARS-CoV-2 Igs by using optical, fluorescence, magnetic and optomagnetic biosensors (reviewed in (Moabelo et al., 2021)) (Figure 4A, *lateral flow tests and colorimetric assays*). Thus, AuNPs have been used in colorimetric assays for detecting target regions on N and RdRp viral genes (Moitra et al., 2020; Kumar et al., 2022) (Figure 4A), or by using quantitative paper-based electrochemical sensor chip to detect the N gene (Alafeef et al., 2020). Moreover, AuNPs allow to detect specific IgM and IgG Abs that recognizes SARS-CoV-2 proteins (S and N) by using a colloidal AuNPs-based lateral-flow assay, where AuNPs are coated with S or N viral proteins to capture Abs (Liu et al., 2020a; Huang et al., 2020b; Peng et al., 2021b) (Figure 4A, *lateral flow tests for viral antigen detection*). AuNPs-based lateral flow tests allow to detect the S or N viral proteins by using S or N protein monoclonal Abs (mAbs) coated to the AuNPs (Mertens et al., 2020; Li et al., 2021a; Liu et al., 2021a), or with sialic acid-coated AuNPs for S protein detection (Baker et al., 2020) (Figure 4A). Likewise, gold nanoislands (AuNIs) have been useful to detect viral genes, such as ORF1ab, RdRp and E by using functional plasmonic photothermal biosensors (Qiu et al., 2020). A real-time optomagnetic biosensor has been applied to detect the RdRp gene by using iron oxide NPs (IONPs) (Tian et al., 2020), whereas graphene oxide (GO) nanosheets, coupled to an anti-S protein Ab, have been used to detect the S antigen in clinical samples by a field-effect transistor (FET)-based biosensing device (Seo et al., 2020). The fluorescence-linked immunoassay technique allows to detect anti-SARS-CoV-2-specific IgG Abs from serum by using MnFe3O4 nanospheres and quantum dot (QD) nanobeads (Guo et al., 2020a). Noteworthy, dye streptavidin coated polymer NPs (SA-DNPs) have been used to detect ORF1ab and N genes by using a multiplex reverse transcription LAMP (mRT-LAMP) coupled with a nanoparticle-based lateral flow biosensor, termed a mRT-LAMP-LFB assay (Zhu et al., 2020d).

These tests are generally low-cost methods useful for PoC facilities and of importance in low-resource settings and regions (De Nardo et al., 2020; Fauziah et al., 2021). Therefore, these genomic, Abs and antigen rapid tests have demonstrated their crucial application for virus surveillance, detection and control of viral outbreaks and community viral transmissions, assisting health systems to rapidly derive only severe ill patients to Hospitals (De Nardo et al., 2020; Mardian et al., 2021) (Figure 4).

## 3 Concluding remarks

The COVID-19 pandemic has demonstrated the relevance of nanomaterials, reagents and technology to combat SARS-CoV-2 infection, community outbreaks and international spreading of the virus and disease. The use of nanotechnology has made possible to detect virus genomic and protein materials from patients’ samples in a rapid, specifically and affordable manner, being able to assume thousands of samples to be tested per day at microbiology hospital departments. This is a task of great magnitude never faced before at region and international levels. Moreover, nanomaterials and derived diagnose techniques (i.e., RT-qPCR and serology test) are responsible for the success of PoC facilities to undertake the mission of virus outbreak control in cities, towns and neighborhoods, and protecting health worker for infection by wearing high-protective PPEs. PoCs have assured rapid and accurate diagnose of infected individuals, helping to derive sever ill patients to the hospital, and assenting public health policies and administrations to make epidemiological and economic decisions.

Although several drugs and Abs have been used for COVID-19 treatment, there are no specific anti-viral treatments available to eradicate SARS-CoV-2 from the organism and revert severe COVID-19 clinical condition in terminal patients (Drożdżal et al., 2021). For this reason, we have decided not to address all the nano-based drug developments and related delivery systems. However, several nanomaterials and technology are candidate to help in the prevention, diagnosis and treatment of SARS-CoV-2 infection and COVID-19 disease. This is the case of some RNA interference (RNAi) formulations to knock-down key viral genes to limit or inhibit viral infection and progression (Idris et al., 2021; Zhang et al., 2022b; Saify Nabiabad et al., 2022). Several *in vitro* studies using these RNAi preparations have obtained promising results against SARS-CoV-2. However, the *in vivo* efficacy of these nanoreagents have been compromised or not yet explored as in clinical trials. Likewise, carbon-based nanomaterials (CBNs) have been developed to analyze their effect on SARS-CoV-2, such as graphene, fullerene, or carbon dots that have been positively assayed on other RNA+ viruses *in vitro* (reviewed in (Serrano-Aroca et al., 2021)). It seems that graphene derivatives with long aliphatic chains limit SARS-CoV-2 replication with some extension, without observing significant cellular toxicity (Donskyi et al., 2021). These CBNs have been tested in other RNA+ viruses with biocompatibility and capacity to induce tissue regeneration, thereby representing promising nanotools for SARS-CoV-2 treatment. Moreover, and considering the antimicrobial effects reported for silver nanoparticles (AgNPs), thought to be associated with the positive charge of silver ions (Ag^+^), some works have explored the inhibition of SARS-CoV-2 infection with AgNPs (Jeremiah et al., 2020). These silver, metal nanoparticles have been assayed in clinical trial as Argovit^®^ (https://clinicaltrials.gov/ct2/show/NCT04894409). The iron oxide nanoparticles (IONPs) Fe_3_O_4_ and Fe_2_O3 have been reported to bind the S viral protein of SARS-CoV-2 (Abo-Zeid et al., 2020). Although there are not clinical studies initiated with these metal IONPs, molecular docking studies point to this possibility. Another metal nanoparticle made of Au-NP, ZnO-NP and ClO_2_ (named TPNT1) has been assayed against SARS-CoV-2. TPNT1 blocks the SARS-CoV-2 viral entry in cells by inhibiting the binding of the S viral protein to the ACE2 receptor (Chang et al., 2021). Noteworthy, the poly (lactic-co-glycolic acid) (PLGA) forms biodegradable polymeric nanoparticles that have been used to deliver several antiviral drugs to treat ill patients suffering the COVID-19 disease, such as Ivermectin (i.e., Ivermectin-loaded PLGA-b-PEG-MAL nanoparticles). PLGA-derived nanoparticles allow to deliver a more potent antiviral dose of the drug and also impairs the S protein/ACE2 binding (Surnar et al., 2020). Several bionanoparticles made of polysaccharides and proteins (i.e., chitosan, alginate, gelatin, and albumin), have been tested or are developed to assay their anti-SARS-CoV-2 activity (reviewed in (Debnath and Srivastava, 2022)). The lack or low toxicity and the excellent biocompatibility showed by all the above-described nanomaterials provide great potential in the development of safe therapeutics based on nanoreagents and technology to prevent SARS-CoV-2 infection and cure the COVID-19 disease.

The use of nanomaterials and technology has reached the major relevance to combat COVID-19 pandemic with the development of nano-based vaccines. In this review, we have addressed the main vaccine designs, prototypes developments and commercial vaccines that have been used to immunize the whole population, in order to protect either individuals at risk or the rest of people. Vaccines have helped to reduce casualties and hospital distress during the last COVID-19 wave. However, these vaccines are not eliciting sterilizing immunity, and vaccinated people could be infected a reinfected by SARS-CoV-2, by the same variant and new evolving emerging variants. The virus could be transmitted between vaccinated people and naturally immunized individuals. Therefore, sterilizing vaccines to immunize at the mucosal, nasal tissues and NALT are a great challenge that nano-based vaccines would be able to achieve in a near future.

Nanotechnology and reagents based on next generation technologies for virus genomic sequencing have similarly achieved a historical milestone, allowing virus surveillance in real time, determining SARS-CoV-2 VOCs and identifying key viral mutations that confer virus resistance against natural- and vaccine-elicited cellular and humoral responses immune escape. This information has been key for making public health decisions for advising vaccine booster shots and wearing masks in public transport, plains, hospitals, schools, bars, companies, and administrations. These genomic sequencing technologies have also been useful in PoC facilities, as nanopore-based approaches have efficiently accomplished, even in low-resources regions. The rapid detection and characterization of new variants is therefore crucial to informing the potential efficacy of vaccines and drugs/treatments.

Altogether, nano-based vaccines and rapid, specific tests for diagnose and outbreak control would help to the efficient control of future COVID-19 epidemic or pandemic episodes. These COVID-19 tools, for sure, will help to the fast development of new and specific nanotools and treatments to challenge future emerging virus and associated crisis that would occur in this XXI century, associated with our global style of life, global warming, and the unsustainable manner we must exploit the resources and biodiversity of our planet.
